# Smart Nanodelivery Systems for Immunometabolic Modulation in Osteoarthritis

**DOI:** 10.1002/exp2.70185

**Published:** 2026-06-08

**Authors:** Qirui Zhao, Zhewen Mi, Shuya Liu, Shijun Liang, Linjia Peng, Zixuan Gao, Jiaming Li, Xiaoqing Lu, Zhiguang Ren, Yongjie Wan, Shengsheng Cui, Longlong Lu, Xiaoyang Gao, Tao Wang, Hui Liang, Furong Tian, de la Fuente Jesus M, Chong Xiang, Luyi Sun, Lichen Xiang, Daxiang Cui

**Affiliations:** ^1^ Department of Orthopedics The First Affiliated Hospital of Henan University Henan University Kaifeng Kaifeng China; ^2^ Henan Province Engineering Technology Research Center of Intelligent Diagnosis and Treatment The First Affiliated Hospital School of Medicine Henan University Kaifeng China; ^3^ Department of Orthopedics Kaifeng Traditional Chinese Medicine Hospital Kaifeng China; ^4^ Key Laboratory of Zhongzhou, Henan Province Kaifeng China; ^5^ Institute of Nano Biomedicine and Engineering School of Sensing Science and Engineering School of Electronic Information and Electrical Engineering Shanghai JiaoTong University Shanghai China; ^6^ Nanolab Research Centre of Technology University Dublin Dublin Ireland; ^7^ Instituto De Nanociencia y Materiales De Aragon CSIC‐University of Zaragoza and CIBER‐BBN Zaragoza Spain; ^8^ Polymer Program Institute of Materials Science and Department of Chemical & Biomolecular Engineering University of Connecticut Storrs USA; ^9^ Division of Intramural Research National Institute of Nursing Research National Institutes of Health Bethesda USA; ^10^ State Key Laboratory of Tribology in Advanced Equipment (SKLT) Tsinghua University Beijing China

**Keywords:** immunometabolic remodelling, osteoarthritis, small‐molecule intervention, smart responsive nanocapsules, synovium‐cartilage axis, targeted delivery systems

## Abstract

Osteoarthritis (OA) is a chronic degenerative joint disease characterised primarily by immunometabolic disorders within the synovium‐cartilage axis. Conventional therapies are limited by poor drug accumulation and nonspecific distribution at lesion sites, resulting in suboptimal and short‐lived efficacy. In recent years, smart responsive nanodelivery systems (SRNSs) have demonstrated considerable potential for OA treatment. This review systematically summarises the major responsive mechanisms of SRNSs—such as pH, reactive oxygen species, enzymes and temperature—and their corresponding targeting strategies, including hyaluronic acid (HA)‐cluster of differentiation 44, arginine‐glycine‐aspartic acid‐collagen II and immune ligand recognition. The dual modulatory roles of SRNSs in the synovium‐cartilage axis are highlighted. By analysing validation evidence from representative material systems—such as zeolitic imidazolate framework‐8, poly(lactic‐co‐glycolic acid) and liposomes—in animal models, we delineate the synergistic mechanisms of SRNSs in inflammation suppression, metabolic remodelling and tissue regeneration. In the discussion section, we further explore key challenges for SRNSs, including biosafety concerns, lesion heterogeneity, manufacturing processes and regulatory standards. Potential strategies—such as biomimetic membrane camouflage, multi‐omics‐based stratification, artificial intelligence (AI) simulation and virtual clinical trials—are also proposed. Additionally, by comparing SRNSs with gene therapy, cell‐penetrating peptides and exosome‐based delivery, this review suggests that future OA therapies may evolve toward hybrid platforms integrating materials, biological systems and gene‐based interventions. Looking ahead, smart systems endowed with self‐feedback, self‐evolution and visualisation capabilities are expected to move OA treatment toward a new era of personalised, adaptive and multidimensional precision interventions.

## Graphical abstract

Smart nanodelivery systems deliver therapeutic payloads across the cartilage matrix to ensure their sustained accumulation within chondrocytes at sites of injury. By targeting specific molecular targets and signaling pathways, these platforms modulate the pathological synovium‐cartilage axis to restore immunometabolic homeostasis and drive tissue repair, thereby effectively treating osteoarthritis and achieving the clinical goal of native joint preservation.

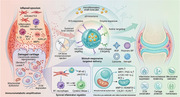

**Abbreviations**
2‐DG: 2‐Deoxy‐D‐Glucose
5‐ASA: 5‐aminosalicylic acid
ACLT: Anterior Cruciate Ligament Transection
ACP: Acid Phosphatase
ADAMTS: A Disintegrin and Metalloproteinase with Thrombospondin Motifs
ADAMTS5: A Disintegrin and Metalloproteinase with Thrombospondin Motifs 5
AI: Artificial Intelligence
AKT: Protein Kinase B
AMPK: Adenosine Monophosphate‐Activated Protein Kinase
ATP: Adenosine Triphosphate
Bax: Bcl‐2‐Associated X Protein
Bcl‐2: B‐Cell Lymphoma 2
CCR6: C‐C Chemokine Receptor Type 6
CD44: Cluster of Differentiation 44
CD86: Cluster of Differentiation 86
CollBP: Type II Collagen‐Binding Peptides
CPPs: Cell‐Penetrating Peptides
DCs: Dendritic Cells
DEX: Dexamethasone
DMF: Dimethyl Fumarate
DMM: Destabilization of the Medial Meniscus
ECM: Extracellular Matrix
FAO: Fatty Acid Oxidation
FLS: Fibroblast‐Like Synoviocytes
GLUT1: Glucose Transporter 1
H_2_O_2_
: Hydrogen Peroxide
HA: Hyaluronic Acid
HIF‐1α: Hypoxia‐Inducible Factor 1‐Alpha
His: Histidine
HK2: Hexokinase 2
HSP90: Heat Shock Protein 90
IFN‐γ: Interferon‐Gamma
IL‐17: Interleukin‐17
IL‐17A: Interleukin‐17A
IL‐1β: Interleukin‐1 Beta
IL‐22: Interleukin‐22
IL‐6: Interleukin‐6
ILC3: Group 3 Innate Lymphoid Cells
iNOS: Inducible Nitric Oxide Synthase
JAK: Janus Kinase
LDHA: Lactate Dehydrogenase A
MMP13: Matrix Metalloproteinase 13
MMPs: Matrix Metalloproteinases
MOFs: Metal‐Organic Frameworks
MRI: Magnetic Resonance Imaging
MSC: Mesenchymal Stem Cell
mTOR: Mechanistic Target of Rapamycin
mTORC1: Mechanistic Target of Rapamycin Complex 1
NF‐κB: Nuclear Factor‐Kappa B
NLRP3: NOD‐Like Receptor Protein 3
Nrf2: Nuclear Factor Erythroid 2‐Related Factor 2
NSAIDs: Nonsteroidal Anti‐Inflammatory Drugs
O_2_^−^
: Superoxide Anions
OA: Osteoarthritis
PBAE: Poly(β‐Amino Ester)
PEG: Polyethylene Glycol
PGC‐1α: Peroxisome Proliferator‐Activated Receptor Gamma Coactivator 1‐Alpha
PI3K: Phosphoinositide 3‐Kinase
PINK1: PTEN‐Induced Kinase 1
PLGA: Poly(Lactic‐Co‐Glycolic Acid)
PPI: Protein‐Protein Interaction
PRP: Platelet‐Rich Plasma
PTT: Photothermal Therapy
RANKL: Receptor Activator of Nuclear Factor Kappa‐Β Ligand
RGD: Arginine‐Glycine‐Aspartic Acid
ROS: Reactive Oxygen Species
scRNA‐seq: Single‐Cell RNA Sequencing
SDH: Succinate Dehydrogenase
SDT: Sonodynamic Therapy
SIRT: Sirtuin
SIRT1: Sirtuin 1
SIRT3: Sirtuin 3
SRNSs: Smart Responsive Nanodelivery Systems
STAT: Signal Transducer and Activator of Transcription
STAT3: Signal Transducer and Activator of Transcription 3
Th17: T Helper 17
TLRs: Toll‐Like Receptors
TNF‐α: Tumor Necrosis Factor‐Alpha
Treg: Regulatory T Cells
VAS: Visual Analogue Scale
VEGF: Vascular Endothelial Growth Factor
WOMAC: Western Ontario and McMaster Universities Osteoarthritis Index
ZIF‐8: Zeolitic Imidazolate Framework‐8
α‐KG: Alpha‐Ketoglutarate

## 1 Introduction

Osteoarthritis (OA) is one of the most prevalent degenerative joint disorders, affecting over 500 million individuals worldwide and imposing a substantial burden on both patients' quality of life and the global healthcare economy [1, 2]. According to reports from the World Health Organisation (WHO) and the global burden of disease study, OA ranks as the 11th leading cause of disability globally, with its prevalence expected to rise steadily by 2050 due to population ageing [1]. In addition to ageing, obesity, joint trauma and genetic predisposition have also been identified as significant risk factors contributing to the earlier onset of OA [3, 4]. Current clinical management relies mainly on symptomatic treatments, including analgesics such as nonsteroidal anti‐inflammatory drugs (NSAIDs) and COX‐2 inhibitors, as well as intra‐articular injections and platelet‐rich plasma (PRP) or mesenchymal stem cell (MSC)‐based therapies. While these approaches alleviate symptoms to some extent, they fail to halt disease progression and are often associated with adverse effects when used long‐term [5, 6]. In advanced stages, joint replacement surgery is commonly performed; however, its use is limited by surgical risks and prolonged recovery periods [5]. Therefore, overcoming the limitations of symptom‐based therapy to achieve early, precise and mechanism‐driven intervention remains a key bottleneck in OA treatment.

**FIGURE 1:**
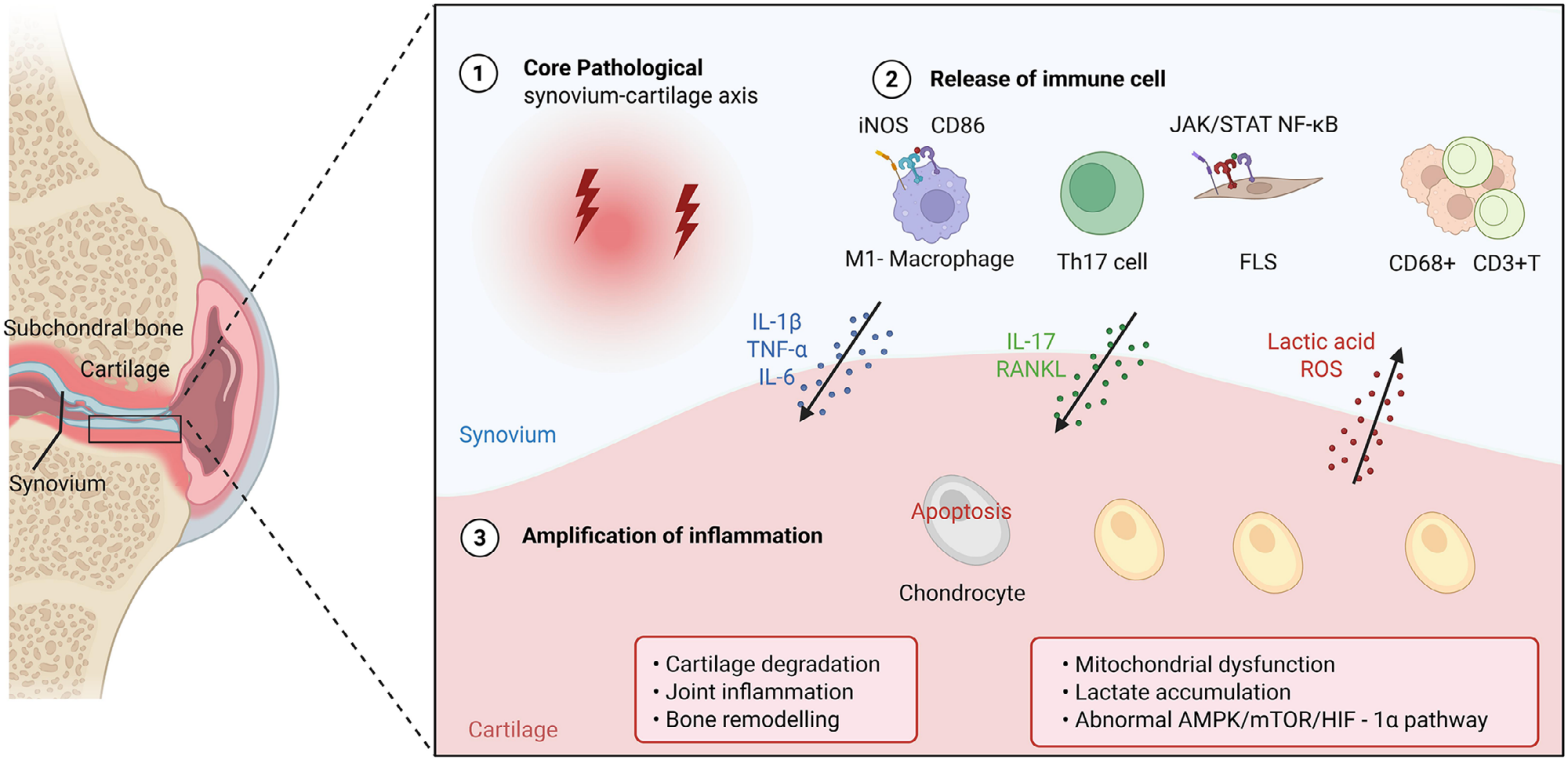
Overview of the structural composition and pathological interactions along the OA synovium‐cartilage axis. *Note*: AMPK, adenosine monophosphate‐activated protein kinase; FLS, fibroblast‐like synoviocytes; HIF‐1α, hypoxia‐inducible factor‐1 alpha; IL‐6, interleukin‐6; IL‐17, interleukin‐17; IL‐1β, interleukin‐1 beta; iNOS: inducible nitric oxide synthase; JAK/STAT, Janus kinase/signal transducer and activator of transcription; Mtor, mechanistic target of rapamycin; NF‐κB: nuclear factor‐kappa B; RANKL, receptor activator of nuclear factor kappa‐B ligand; ROS, reactive oxygen species; TNF‐α: tumor necrosis factor‐alpha.

The traditional view often attributes OA to the ‘wear and tear’ of articular cartilage caused by long‐term mechanical loading, with research primarily focusing on cartilage matrix degradation and mechanical imbalance. However, accumulating evidence from molecular biology and imaging studies has gradually reshaped this paradigm. OA is no longer considered a disease of isolated cartilage degeneration; rather, it is a chronic whole‐joint disorder involving complex interactions among multiple tissues, including the synovium, cartilage and subchondral bone. Low‐grade inflammation and synovitis are now recognised as important pathological components of the disease [7, 8, 9] (Figure 1). Within this conceptual framework, the ‘synovium‐cartilage axis’ has been proposed as a critical hub driving OA progression. Immune cells and synovial fibroblasts within the synovium continuously release inflammatory mediators that induce chondrocyte apoptosis and matrix degradation. Meanwhile, chondrocytes exposed to inflammatory and mechanical stress develop mitochondrial dysfunction and metabolic reprogramming, which further amplifies inflammatory responses, forming a bidirectional amplification loop between immune signalling and metabolic dysregulation [8, 9, 10]. This ‘immunometabolic axis’ model is also supported by clinical association evidence. The longitudinal multicentre osteoarthritis study cohort demonstrated that MRI‐detected joint effusion‐synovitis, even when present in knees without radiographic OA, predicts subsequent tibiofemoral cartilage loss over 30 months, suggesting that synovitis may serve as a preceding signal of structural progression [11]. Contrast‐enhanced MRI studies further show that the severity of synovitis is significantly associated with radiographic disease severity and widespread cartilage damage [12]. Consistent with imaging findings, biomarkers related to inflammation and matrix turnover in synovial fluid or peripheral blood can characterise inflammatory phenotypes and correlate with clinical symptoms or imaging severity. Some inflammation‐associated products, such as CRPM, are also linked to cartilage degradation processes [13, 14]. At the structural outcome level, urinary CTX‐II combined with serum hyaluronic acid and other biomarkers can predict structural progression, including joint space narrowing [15]. Taken together, the paradigm shift from a ‘wear‐and‐tear’ model to one centred on immunometabolic coupling imbalance provides a more testable and translational framework for mechanistic stratification and targeted therapeutic intervention in OA.

Beyond cytokine‐driven inflammation, OA joints exhibit broader disturbances in metabolic homeostasis, characterised by an imbalance between anabolic and catabolic processes, reprogramming of energy metabolism and disruption of redox homeostasis. Chondrocytes shift from a homeostatic maintenance state toward a catabolic phenotype, with decreased expression of anabolic genes such as COL2A1 and ACAN, while degradative enzymes, including matrix metalloproteinase‐13 (MMP‐13) and a disintegrin and metalloproteinase with thrombospondin motifs‐5 (ADAMTS‐5), remain persistently upregulated, resulting in continuous net loss of extracellular matrix [16]. In terms of energy metabolism, OA chondrocytes may undergo Warburg‐like metabolic reprogramming characterised by a shift from oxidative phosphorylation toward glycolysis, which is closely coupled with signalling pathways such as HIF‐1α, mTOR and AMPK [17, 18, 19, 20]. Concurrently, mitochondrial dysfunction and abnormalities in lipid and amino acid metabolism further exacerbate cellular stress and contribute to the persistence of inflammation [21, 22]. At the redox level, excessive production of reactive oxygen species (ROS), together with insufficient NRF2‐mediated antioxidant defence, triggers oxidative stress and lipid peroxidation, leading to chondrocyte apoptosis or ferroptosis and promoting matrix degradation [23, 24]. Together, these metabolic abnormalities amplify synovial inflammation and create a broader picture of immunometabolic imbalance along the synovium‐cartilage axis.

To address the immunometabolic disturbances described above, small molecules such as resveratrol, triptolide and metformin have been shown to suppress synovial inflammation and chondrocyte apoptosis by activating AMPK or inhibiting NF‐κB, thereby reducing ROS production and improving mitochondrial function [21, 25, 26]. However, most currently available agents primarily target inflammatory endpoints or single signalling pathways, making it difficult to simultaneously and durably correct key metabolic abnormalities such as shifts in energy metabolism and oxidative stress. In addition, maintaining effective intra‐articular exposure is difficult because these agents are readily degraded or inactivated by the cartilage matrix barrier and the synovial fluid microenvironment. Their clinical translation, therefore, remains constrained by limitations in bioavailability, targeting specificity and systemic toxicity [27]. Accordingly, smart responsive nanodelivery systems (SRNSs) have been developed to exploit lesion‐associated signals, such as acidic pH, elevated ROS levels, or specific enzyme activity, in order to achieve locally triggered drug release and targeted accumulation. These systems also support co‐delivery or sequential delivery strategies, thereby enabling more integrated immunometabolic intervention. Platforms such as PLGA, ZIF‐8 and polymeric micelles have already been used to deliver relevant small molecules and to ameliorate abnormalities at the synovium‐cartilage interface [27].

In summary, the pathological progression of OA stems from immunometabolic imbalance within the synovium‐cartilage axis. Smart responsive nanocarriers, through local environmental sensing and targeted drug delivery, offer a promising approach to overcoming the limitations of small‐molecule therapies. This review systematically examines the pathological role of immunometabolic dysregulation along the synovium‐cartilage axis in OA. It further focuses on the intervention mechanisms of anti‐inflammatory small molecules and the structure and application of SRNSs, highlighting integrated advances in ‘mechanism‐carrier‐therapeutic efficacy’ to provide both theoretical and engineering foundations for disease‐modifying treatment of OA.

## 2 Immunometabolic Dysregulation of the Synovium‐Cartilage Axis Reveals New Mechanisms Underlying OA Progression

### 2.1 Synovial Inflammation and Immune Lineage Remodelling

**FIGURE 2:**
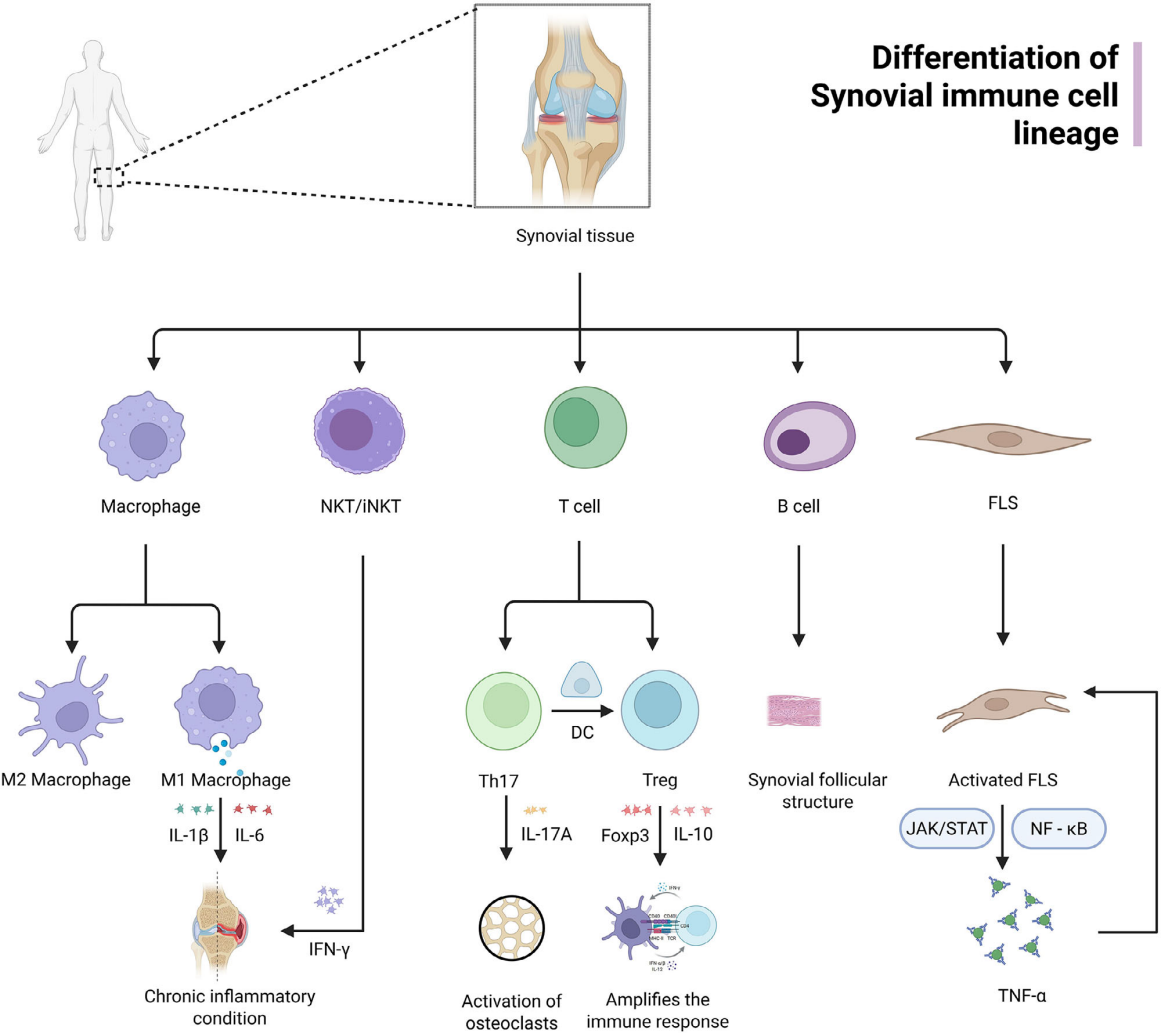
Immune cell lineage dynamics and proinflammatory cytokine release in the synovium. *Note*: DC, dendritic cell; FLS, fibroblast‐like synoviocyte; Foxp3, forkhead box P3; IL‐1β, interleukin‐1 beta; IL‐6, interleukin‐6; IL‐17A, interleukin‐17A; JAK/STAT, Janus kinase/signal transducer and activator of transcription; M1 macrophage, classically activated macrophage; M2 Macrophage, alternatively activated macrophage; NF‐κB, nuclear factor kappa‐B; NKT/ iNKT, natural killer T/invariant natural killer T; TNF‐α, tumor necrosis factor‐alpha; Treg, regulatory T cell; Th17, T helper 17 cell.

Synovial tissue plays a central role in both the initiation and maintenance of inflammation in OA [10, 28]. In healthy joints, the synovium is composed primarily of quiescent macrophages and fibroblast‐like synoviocytes (FLSs), which together maintain joint lubrication and immune homeostasis (Figure 2) [29]. In OA patients, the synovium exhibits hallmark pathological features such as inflammatory hyperplasia, neovascularisation, and immune cell infiltration, underscoring its importance in disease progression [28, 30]. Macrophages are markedly increased in OA synovium and tend to polarise toward the pro‐inflammatory M1 phenotype, characterised by the expression of markers such as inducible nitric oxide synthase (iNOS) and cluster of differentiation 86 (CD86). These macrophages secrete inflammatory cytokines, including interleukin‐1 beta (IL‐1β), tumour necrosis factor‐alpha (TNF‐α) and interleukin‐6 (IL‐6), which collectively promote persistent local inflammation [10, 31].

FLSs also undergo phenotypic remodelling in the disease state. Activated FLS adopt an aggressive phenotype and continuously secrete pro‐inflammatory mediators through activation of the Janus kinase (JAK)/signal transducer and activator of transcription (STAT) and NF‐κB signalling pathways, forming a self‐reinforcing positive feedback loop [29]. Additionally, adaptive immunity contributes to the maintenance and amplification of OA synovitis. Flow cytometric analyses of synovial tissue and synovial fluid from patients with knee OA have demonstrated marked local infiltration of CD4 + T cells within the joint. Enrichment of Th17‐like cells or their associated markers, such as CD161 + C‐C chemokine receptor type 6 (CCR6)+, has also been observed in the synovium or synovial fluid and their levels correlate with clinical outcomes, including radiographic severity, pain and functional limitation [32, 33]. In contrast, the proportion and functional status of regulatory T cells (Treg) infiltrating the joint are closely associated with symptom burden and insufficient Treg‐mediated counter‐regulation may weaken the suppression of inflammatory amplification [34]. Meanwhile, IL‐17A levels in synovial fluid and serum are positively correlated with Kellgren–Lawrence grade, pain scores and functional assessment scores, providing humoral evidence for the involvement of the Th17 axis in OA [35]. Taken together, these findings suggest that OA is characterised by an immune imbalance marked by enhanced Th17 responses and insufficient Treg‐mediated regulation, rather than simply extrapolating this concept from the RA paradigm.

In recent years, single‐cell RNA sequencing (scRNA‐seq) has been employed to analyse the cellular heterogeneity of synovial tissue in OA, revealing several functionally distinct subpopulations. These include pro‐inflammatory FLS characterised by high IL‐6 and low CD55 expression (IL6^high^/CD55^low^) and anti‐inflammatory macrophage subsets marked by TREM2 expression. This refined cellular atlas has deepened our understanding of the synovial inflammatory network and provided cell‐level targets for precision drug delivery [36]. Moreover, in OA models such as destabilisation of the medial meniscus (DMM) in mice and anterior cruciate ligament transection (ACLT) in rabbits, increases in synovial macrophage numbers and inflammatory cytokine expression have been closely associated with joint functional deterioration, further validating their pathological roles [37, 38].

Surgically induced models, such as DMM and ACLT, are accompanied by a pronounced acute injury response during the early phase after model induction. Synovial macrophage infiltration and increased cytokine production partly reflect postoperative inflammatory fluctuations. Therefore, when extrapolating findings from these models to human primary, age‐related OA, it is necessary to integrate evidence from spontaneous and naturally ageing models. For example, STR/ort spontaneous OA mice develop synovial hyperplasia associated with cartilage damage even in the absence of surgical induction, accompanied by low‐grade inflammation and elevated markers of oxidative stress, including RAGE and its ligands AGE and HMGB1, along with increased levels of pro‐inflammatory cytokines in serum [39]. In naturally aged mice, synovial macrophages may exhibit features of immunosenescence with a bias toward the M1 phenotype, leading to enhanced secretion of SASP factors that occur in parallel with cartilage degeneration. These findings suggest that immune activation can also function as an endogenous driver of ageing‐related OA [40]. Furthermore, in spontaneous or age‐associated OA models such as Dunkin Hartley guinea pigs, early interventions targeting the infrapatellar fat pad‐synovium complex can alter the trajectory of disease progression, further supporting the role of the synovial immune microenvironment in sustaining OA progression even in non‐traumatic contexts [41, 42]. In this section, we incorporate a discussion of the translational relevance and limitations of these models to avoid misinterpreting acute injury‐induced stress responses as mechanisms specific to primary OA.

Beyond these core immune subpopulations, a range of immunomodulatory cells also contribute to the inflammatory network in OA. CD8^+^ T cells secrete interferon‐gamma (IFN‐γ) and granzyme B to induce chondrocyte apoptosis. B cells form follicle‐like structures and release autoantibodies that activate the complement system. Dendritic cells (DCs) regulate the differentiation of Th17 and Treg cells through antigen presentation. Natural killer T (NKT) and invariant NKT (iNKT) cells and Group 3 innate lymphoid cells modulate the synovium‐cartilage barrier and early inflammatory responses via IFN‐γ and interleukin‐17 (IL‐17)/interleukin‐22 (IL‐22), respectively [43, 44]. Although these ‘auxiliary cell populations’ do not directly initiate inflammation, their synergistic interactions with core immune units play indispensable roles in amplifying chronic inflammation, exacerbating immunometabolic imbalance and promoting bone destruction in OA.

### 2.2 Cartilage Metabolic Imbalance and Mitochondrial Dysfunction

**FIGURE 3:**
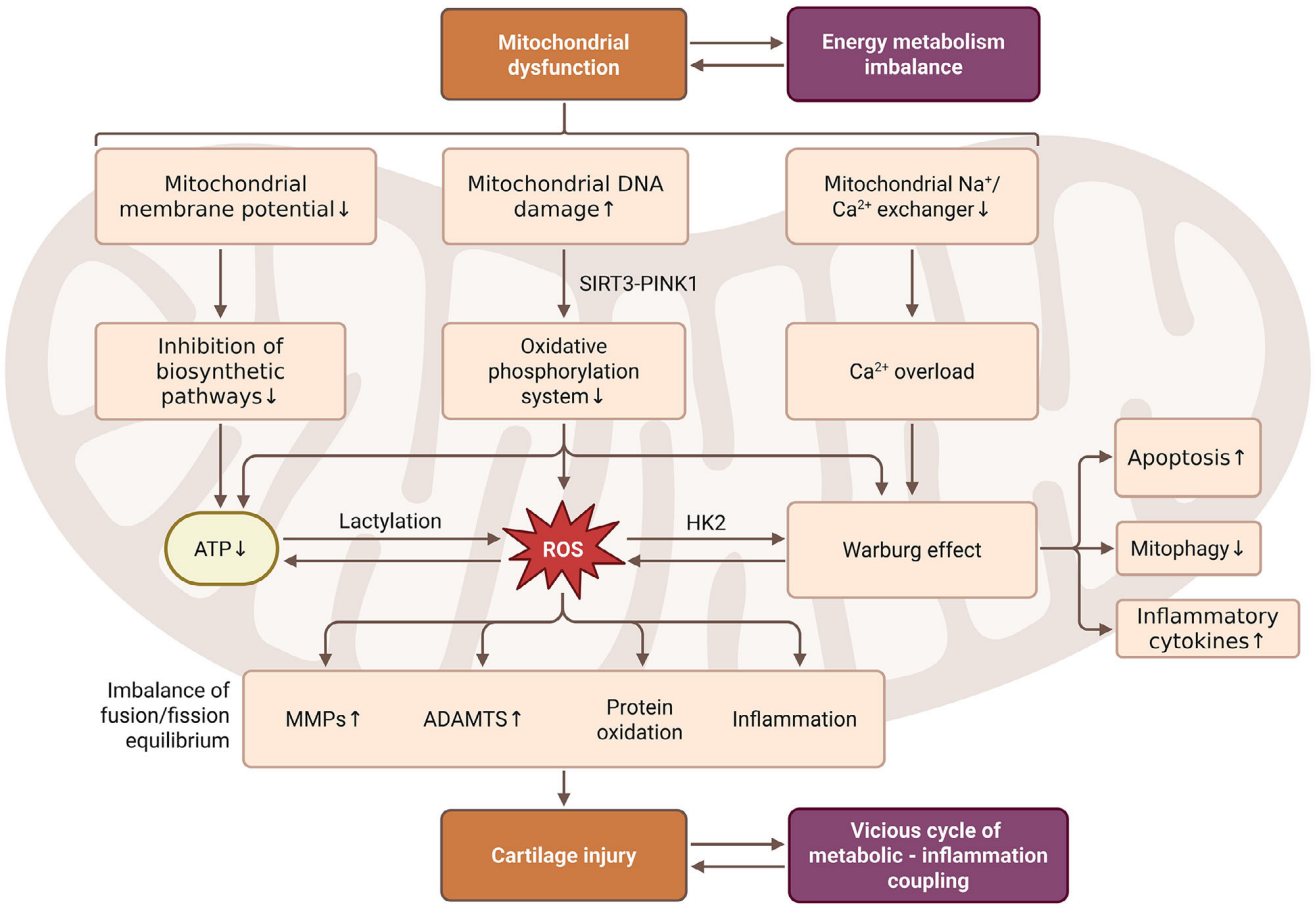
Mechanisms of mitochondrial dysfunction and energy metabolism imbalance in OA cartilage cells. *Note*: ADAMTS, a disintegrin and metalloproteinase with thrombospondin motifs; ATP, adenosine triphosphate; HK2, hexokinase 2; MMPs, matrix metalloproteinases; OGD/R, oxygen‐glucose deprivation/reoxygenation; PINK1, PTEN‐induced putative kinase 1; ROS, reactive oxygen species; SIRT3, sirtuin 3.

Another central feature of OA pathology is the metabolic reprogramming of chondrocytes [45]. In healthy cartilage, chondrocytes primarily rely on glycolysis to sustain the synthesis of type II collagen and proteoglycans. However, under conditions of inflammation, hypoxia and mechanical stress, their metabolic profile is markedly altered, characterised by suppressed mitochondrial oxidative phosphorylation, excessive ROS production and abnormally enhanced glycolysis (Figure 3) [45, 46, 47].

Mitochondrial dysfunction is considered a key driver of cartilage degeneration. In OA chondrocytes, mitochondrial membrane potential is diminished, adenosine triphosphate (ATP) synthesis is impaired and DNA damage and suppressed biosynthetic activity are commonly observed [48]. Energy metabolism shifts toward a Warburg‐like glycolytic phenotype, leading to lactate accumulation. This, in turn, inhibits ECM synthesis and upregulates catabolic enzymes such as matrix metalloproteinases (MMPs) and a disintegrin and metalloproteinase with thrombospondin motifs (ADAMTS), forming a self‐reinforcing ‘lactate‐inflammation’ amplification loop [45, 47]. Moreover, excessive ROS production further promotes chondrocyte apoptosis and activates inflammatory signalling pathways, establishing a vicious cycle of metabolic and inflammatory dysregulation [49].

Both in vivo and in vitro studies have validated this mechanism. In DMM and ACLT models, the extent of mitochondrial dysfunction was positively correlated with cartilage degradation. Treatment with mitochondrial‐targeted antioxidants such as MitoQ or SS‐31 reversed degenerative changes and reduced inflammatory responses. Co‐culture experiments of FLS and chondrocytes demonstrated that TNF‐α and IL‐1β disrupted cartilage metabolic homeostasis via the ROS/hypoxia‐inducible factor 1‐alpha (HIF‐1α) pathway, suggesting the existence of metabolic crosstalk between the synovium and cartilage [50]. Metabolomic analyses further confirmed significant abnormalities in key metabolites such as lactate and pyruvate within OA cartilage [51].

Recent studies have uncovered several critical molecular mechanisms. Lactate‐induced lysine lactylation was found to activate MMP13 expression, thereby exacerbating ECM degradation. Downregulation of the mitochondrial deacetylase sirtuin 3 (SIRT3) impaired oxidative phosphorylation and promoted ROS accumulation. Upregulation of the glycolytic rate‐limiting enzyme hexokinase 2 (HK2) enhanced lactate production through activation of the mechanistic target of rapamycin (mTOR)‐HIF‐1α signalling pathway [52, 53, 54]. These findings collectively indicate that maintaining mitochondrial function and regulating metabolic pathways represent promising therapeutic targets for preventing cartilage degeneration in OA.

### 2.3 Immunometabolic Crosstalk Pathways and Signalling Networks

**FIGURE 4:**
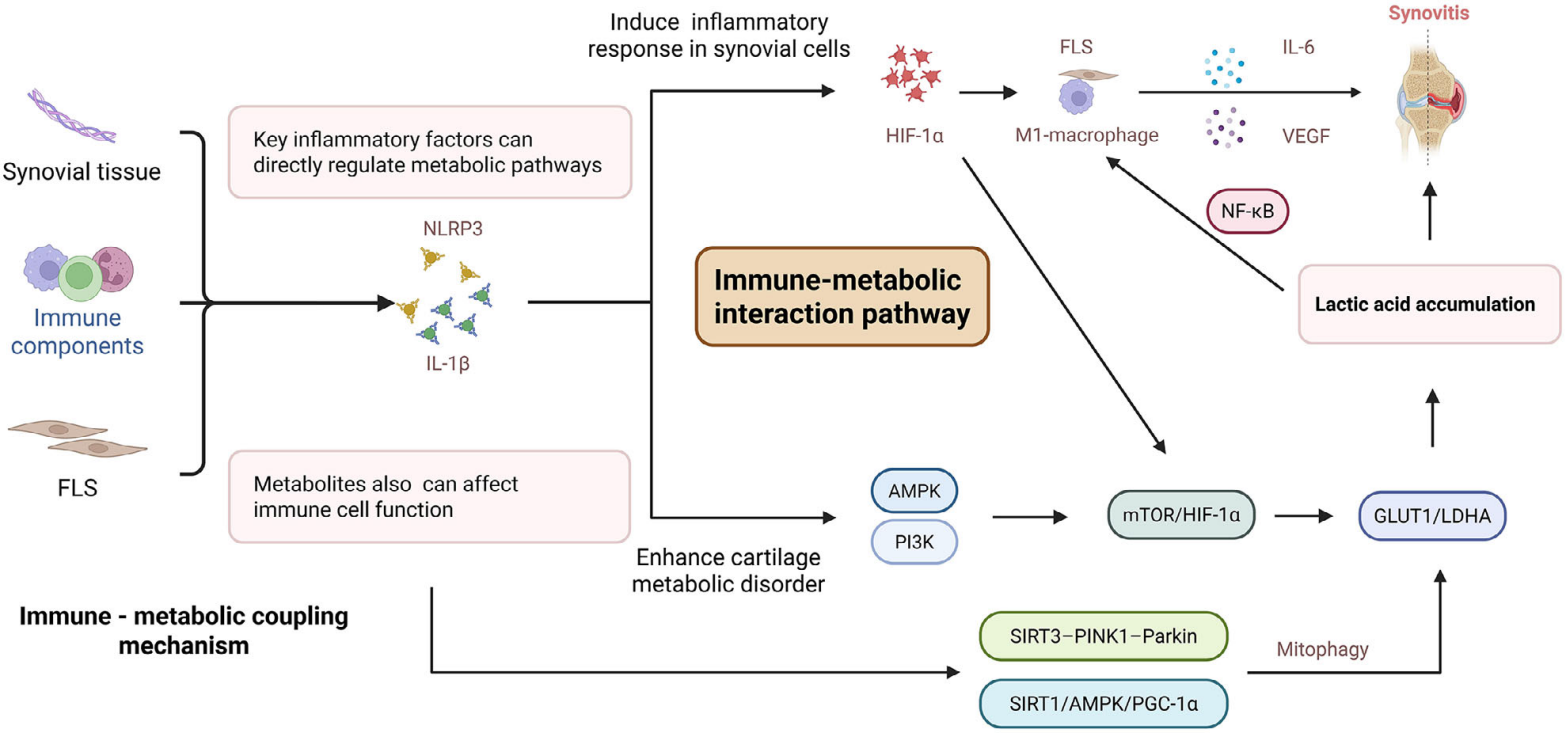
Schematic illustration of immunometabolic interactions along the synovium‐cartilage axis in OA. *Note*: AMPK, adenosine monophosphate‐activated protein kinase; FLS, fibroblast‐like synoviocytes; GLUT1, glucose transporter 1; HIF‐1α, hypoxia‐inducible factor‐1 alpha; IL‐1β, interleukin‐1 beta; LDHA, lactate dehydrogenase A; mTOR, mechanistic target of rapamycin; NF‐κB, nuclear factor kappa‐B; NLRP3, NOD‐like receptor protein 3; PI3K, phosphatidylinositol 3‐kinase; PINK1, PTEN‐induced putative kinase 1; PGC‐1α, peroxisome proliferator‐activated receptor gamma coactivator 1 alpha; SIRT3, sirtuin 3; VEGF, vascular endothelial growth factor.

In OA synovial and cartilage tissues, immune responses and metabolic activity are tightly coupled (Figure 4) [55]. Key inflammatory cytokines can directly regulate metabolic pathways, while metabolic intermediates can, in turn, influence immune cell function via specific receptors or signalling cascades [56].

The IL‐1β/HIF‐1α/mTOR axis is one of the best‐characterised crosstalk pathways. IL‐1β suppresses AMPK while activating mTOR and HIF‐1α signalling, thereby enhancing glycolysis and exacerbating metabolic dysfunction in cartilage [54, 57, 58]. Under hypoxic conditions, HIF‐1α not only promotes the secretion of inflammatory mediators such as IL‐6 and vascular endothelial growth factor (VEGF) by FLS and macrophages, but also regulates glucose transporter 1 (GLUT1) expression and lactate production in chondrocytes [56, 57, 59]. In the inflammatory microenvironment of the synovium, sustained activation of HIF‐1α leads to persistent upregulation of mTOR signalling, ultimately driving chronic immune cell activation and the disruption of local metabolic homeostasis [58]. In addition to this pathway, the AMPK‐mTOR‐HIF‐1α axis constitutes a metabolic feedback loop that senses energy stress and helps maintain homeostasis under inflammatory conditions. Moreover, the phosphoinositide 3‐kinase (PI3K)/protein kinase B (AKT)/mTOR/STAT3 axis coordinately regulates cell survival, the expression of glycolytic enzymes and the invasive behaviour of synoviocytes, playing a pivotal role in synovial remodelling and cellular viability during the early and intermediate stages of OA progression [54, 57, 59].

Metabolic intermediates also feed back into immune pathways. Lactate activates NF‐κB signalling via its receptor G protein‐coupled receptor 81 (GPR81), promoting M1 macrophage polarisation and enhancing the inflammatory response of FLS. Succinate induces ROS production through succinate dehydrogenase, thereby activating the NOD‐like receptor protein 3 (NLRP3) inflammasome and facilitating the maturation and release of IL‐1β. In contrast, citrate and alpha‐ketoglutarate (α‐KG) participate in epigenetic modifications that regulate immune cell metabolism and inflammatory gene transcription [46, 56, 60]. These metabolic signals and inflammatory mediators form a bidirectional amplification loop that accelerates local immunometabolic imbalance in OA.

Metabolic reprogramming of immune cells represents the cellular basis of this crosstalk network. M1 macrophages rely heavily on glycolysis, whereas M2 macrophages primarily depend on fatty acid oxidation (FAO) [61]. Similarly, Th17 cell activation depends on the mTOR complex 1 (mTORC1) pathway, while Treg cells favour lipid metabolism [58]. These metabolic phenotypes dictate immune cell polarisation and functional states, highlighting the potential for dual‐targeted interventions that modulate both energy metabolism and immune homeostasis [62].

Concurrently, mitochondrial stress and autophagy‐regulating pathways play protective roles in maintaining immunometabolic stability. The sirtuin 1 (SIRT1)/AMPK/peroxisome proliferator‐activated receptor gamma coactivator 1‐alpha (PGC‐1α) axis promotes mitochondrial biogenesis and autophagic flux, thereby reducing oxidative stress and restoring energy balance. The SIRT3‐PTEN‐induced kinase 1 (PINK1)‐Parkin pathway facilitates mitophagy, eliminating damaged mitochondria and limiting ROS accumulation, which in turn alleviates apoptosis in synovial and cartilage cells [45, 46, 63]. These ‘metabolic buffering pathways’ serve as intrinsic counter‐regulatory mechanisms within the inflammatory amplification cascade.

In summary, the immunometabolic crosstalk within the OA synovium‐cartilage axis constitutes a complex network orchestrated by inflammatory signals, metabolic products and energy stress responses. Immune activation drives metabolic reprogramming, while metabolic imbalance reciprocally intensifies inflammatory responses. A comprehensive understanding and targeted modulation of this network may enable multi‐tiered therapeutic strategies encompassing immune regulation, metabolic intervention and structural protection, thereby offering a systematic framework for precision treatment of OA.

### 2.4 Experimental Validation of Immunometabolic Mechanisms and Target Mapping for Small‐Molecule Intervention

Immuno‐metabolic dysregulation along the synovium‐cartilage axis has been extensively validated in both clinical specimens and animal models as a key driving force in OA progression. Clinically, elevated expression levels of IL‐1β, TNF‐α, HIF‐1α and GLUT1 have been detected in the synovial tissue of OA patients, showing a positive correlation with pain severity (visual analogue scale [VAS]) and functional impairment (Western Ontario and McMaster Universities Osteoarthritis Index) scores [64, 65]. Notably, GLUT1—a critical glucose transporter in glycolysis—has been consistently upregulated in both synovium and cartilage of OA patients. Its co‐expression with glycolytic enzymes such as lactate dehydrogenase A (LDHA) forms a ‘metabolic activation signature,’ suggesting a strong link between enhanced glucose metabolism and inflammatory amplification [66].

Imaging evidence further supports this pathological process. Arthroscopic and magnetic resonance imaging (MRI) analyses have demonstrated that synovial thickening and neovascularisation are significantly associated with subchondral bone sclerosis and cartilage degeneration [67]. Recent advances in MRI combined with radiomics analysis (MRI‐radiomics) have shown that parameters such as synovial thickness, perfusion velocity and signal texture features can sensitively reflect early neovascular activity and correlate with the rate of OA progression.

Animal studies have provided a more systematic platform for mechanistic exploration. In classical OA models such as DMM in mice and ACLT in rabbits, synovial inflammation and cartilage degradation were observed to occur concurrently. Interventions with metabolic modulators (e.g., metformin, 2‐deoxy‐D‐glucose [2‐DG]) or immunosuppressants (e.g., anti‐IL‐1β antibodies) significantly delayed cartilage degeneration, indicating a strong pathological interdependence between these two processes [68]. In addition, emerging methodologies such as synovium‐cartilage co‐culture systems, FLS‐chondrocyte exosome assays, scRNA‐seq and integrated metabolomic analyses have further advanced our understanding of the molecular interplay between synovial and cartilage tissues [69]. To better elucidate the key molecular nodes within immune and metabolic pathways of the synovium‐cartilage axis and to identify potential targets for small‐molecule intervention, this study summarises the major validated targets and representative compounds from current experimental and clinical research (Table 1) [58], [70, 71, 72, 73].

**TABLE 1:** Key mechanisms and representative small‐molecule interventions in the synovium‐cartilage axis of osteoarthritis.

Mechanism category	Key molecules/pathways	Experimental and clinical evidence	Representative small‐molecule interventions
Synovial immune activation [70]	IL‐1β/NF‐κB/JAK‐STAT	Elevated proportions of pro‐inflammatory cells (M1 macrophages, Th17 cells) in OA synovium; increased IL‐1β and TNF‐α expression; inhibition of this axis alleviates synovial hyperplasia	Celastrol, fluticasone, resveratrol
Chondrocyte metabolic Dysregulation [58]	mTOR/HIF‐1α/ROS pathways	Upregulation of HIF‐1α and GLUT1 in chondrocytes; mitochondrial dysfunction; mTOR inhibition restores metabolic homeostasis	Rapamycin, MitoQ, SS‐31
Metabolism‐immunity feedback [71]	Lactate‐GPR81/NLRP3/ROS axis	Synovial lactate accumulation and elevated ROS; blockade of metabolic signaling reduces IL‐1β expression	Metformin, 2‐DG, celastrol
Energy homeostasis reconstruction [72]	SIRT1‐AMPK‐PGC‐1α/SIRT3‐PINK1‐Parkin axis	Mitochondrial protectants restore ATP production and suppress inflammation in animal models	MitoQ, resveratrol, metformin
Integrated immunometabolic balance [73]	Th17/Treg and M1/M2 polarisation regulation	Th17/Treg imbalance observed in OA synovium; small‐molecule modulation restores immune homeostasis	Metformin, celastrol

In conclusion, OA should no longer be viewed as a disease of a single tissue, but rather as a systemic disorder driven by the coordinated dysregulation of inflammation and metabolism along the synovium‐cartilage axis. Identifying and modulating immunometabolic abnormalities within this axis has not only become a focal point in fundamental research but also lays a theoretical foundation for personalised clinical therapies.

## 3 Immunometabolic Effects and Mechanisms of Small‐Molecule Drugs

### 3.1 Classification and Principal Pathways of Action

Anti‐inflammatory small‐molecule drugs have emerged as a major focus in OA treatment research due to their structural stability, ease of synthesis and broad regulatory effects across multiple signalling pathways [74]. Based on their origin and mechanisms of action, these small molecules are commonly categorised into three types: naturally derived compounds, synthetic derivatives and repurposed drugs.

Naturally derived small molecules are primarily sourced from botanical and traditional Chinese medicinal compounds, including resveratrol, curcumin, quercetin and berberine [75, 76, 77, 78]. Their major advantages are relatively low toxicity and multitarget regulatory properties, often simultaneously modulating pathways associated with inflammation, oxidative stress and metabolic homeostasis (Table 2). Table 2 summarises the principal pathway targets, levels of evidence and key readout indicators of representative small molecules, supporting the importance of these core pathways in OA intervention [78, 79, 80, 81, 82, 83, 84].

**TABLE 2:** Natural small‐molecule drugs and their mechanisms in osteoarthritis.

Category	Representative drug	Major target pathway	Primary cellular effects
Natural extracts	Resveratrol [79]	SIRT1/AMPK/NF‐κB	Inhibits Th17 differentiation, promotes Treg restoration, improves mitochondrial function
	Curcumin [80]	MAPK/PI3K‐Akt/NF‐κB	Antioxidant, anti‐apoptotic, suppresses MMP‐13 expression
	Quercetin [81]	PI3K‐Akt/SIRT1	Restores immune balance, alleviates oxidative stress
	Berberine [78]	AMPK‐mTOR/TLR4	Regulates glucose‐lipid metabolism, suppresses synovial inflammation
Synthetic derivatives	Metformin [82]	AMPK‐mTOR/SIRT3	Inhibits pro‐inflammatory cytokines, restores mitochondrial function, enhances M2 macrophage polarization
	Tofacitinib [82]	JAK‐STAT3	Reduces synovial infiltration and angiogenesis
Drug repositioning	Dexamethasone [83]	NF‐κB/NLRP3	Inhibits pro‐inflammatory cytokines and ROS production
	Dimethyl fumarate (DMF) [84]	Nrf2/NF‐κB	Activates antioxidant genes and reduces inflammation

In contrast, synthetic small‐molecule derivatives are generally developed through targeted modification of specific signalling pathways. For example, metformin, an AMPK activator, can modulate inflammation and energy homeostasis through the AMPK/mTOR signalling axis [85]. Tofacitinib, a JAK inhibitor, blocks JAK/STAT3 signalling and has demonstrated anti‐inflammatory potential in inflammatory arthritis as well as in OA animal models [86].

**FIGURE 5:**
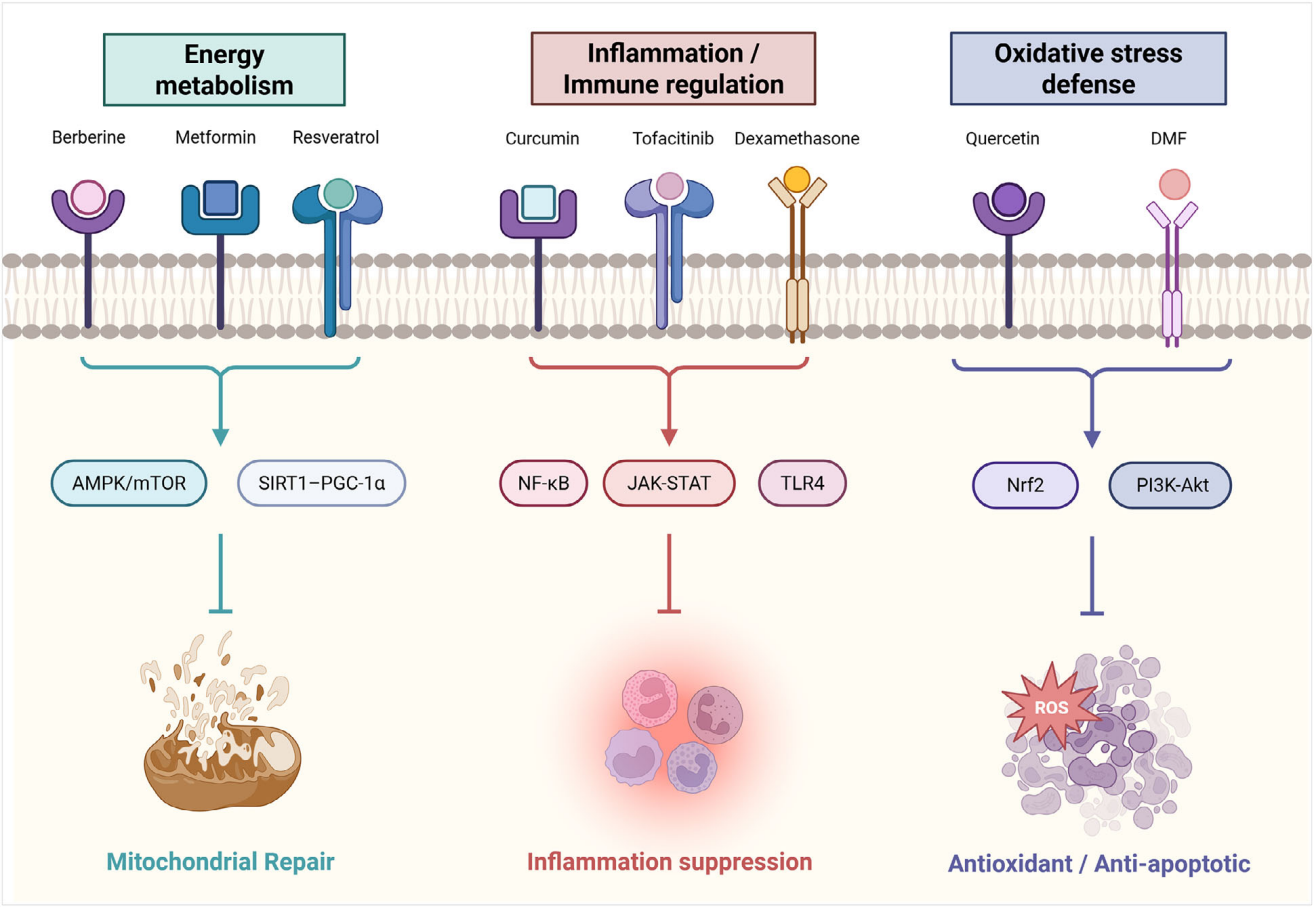
Signaling targets and mechanistic pathways of anti‐inflammatory small‐molecule drugs. *Note*: Akt, protein kinase B; AMPK, adenosine monophosphate‐activated protein kinase; DMF, dimethyl fumarate; IL‐1β: interleukin‐1 beta; JAK, Janus kinase; mTOR, mechanistic target of rapamycin; Nrf2, nuclear factor erythroid 2‐related factor 2; NF‐κB, nuclear factor kappa‐light‐chain‐enhancer of activated B cells; PGC‐1α, peroxisome proliferator‐activated receptor gamma coactivator 1‐alpha; PI3K, phosphoinositide 3‐kinase; ROS, reactive oxygen species; SIRT1, sirtuin 1; SIRT3, sirtuin 3; STAT, signal transducer and activator of transcription; TNF‐α, tumor necrosis factor‐alpha.

Drug repurposing candidates include agents such as dexamethasone and dimethyl fumarate (DMF). These drugs were originally developed for immune‐mediated or metabolic disorders; however, their ability to inhibit NF‐κB signalling and the NLRP3 inflammasome, or to activate the Nrf2‐mediated antioxidant pathway, has revealed potential for repositioning in OA by contributing to both inflammatory control and metabolic restoration [84]. It should be emphasised that although different classes of small molecules vary in origin and pharmacological positioning, their therapeutic efficacy often manifests through the coordinated regulation of three major mechanistic axes within the synovium‐cartilage axis: immune responses, metabolic regulation and intercellular communication. Collectively, these studies indicate that small‐molecule drugs can achieve dual modulation of inflammation and metabolism along the synovium‐cartilage axis, largely through the coordinated regulation of multiple signalling pathways (Figure 5). Figure 5 summarises these pathway nodes and their interactive relationships.

### 3.2 Multi‐Target Mechanistic Exploration of Small‐Molecule Drugs in OA

The therapeutic effects of small‐molecule drugs in OA can be attributed to three principal mechanistic axes: immunometabolic remodelling, mitochondrial energy restoration and modulation of synovium‐cartilage intercellular signalling. Together, these form an integrated ‘immune‐metabolism‐structure’ intervention network.

#### 3.2.1 Immune Axis: Reducing Synovial Inflammatory Burden and Immune Amplification Loops

**FIGURE 6:**
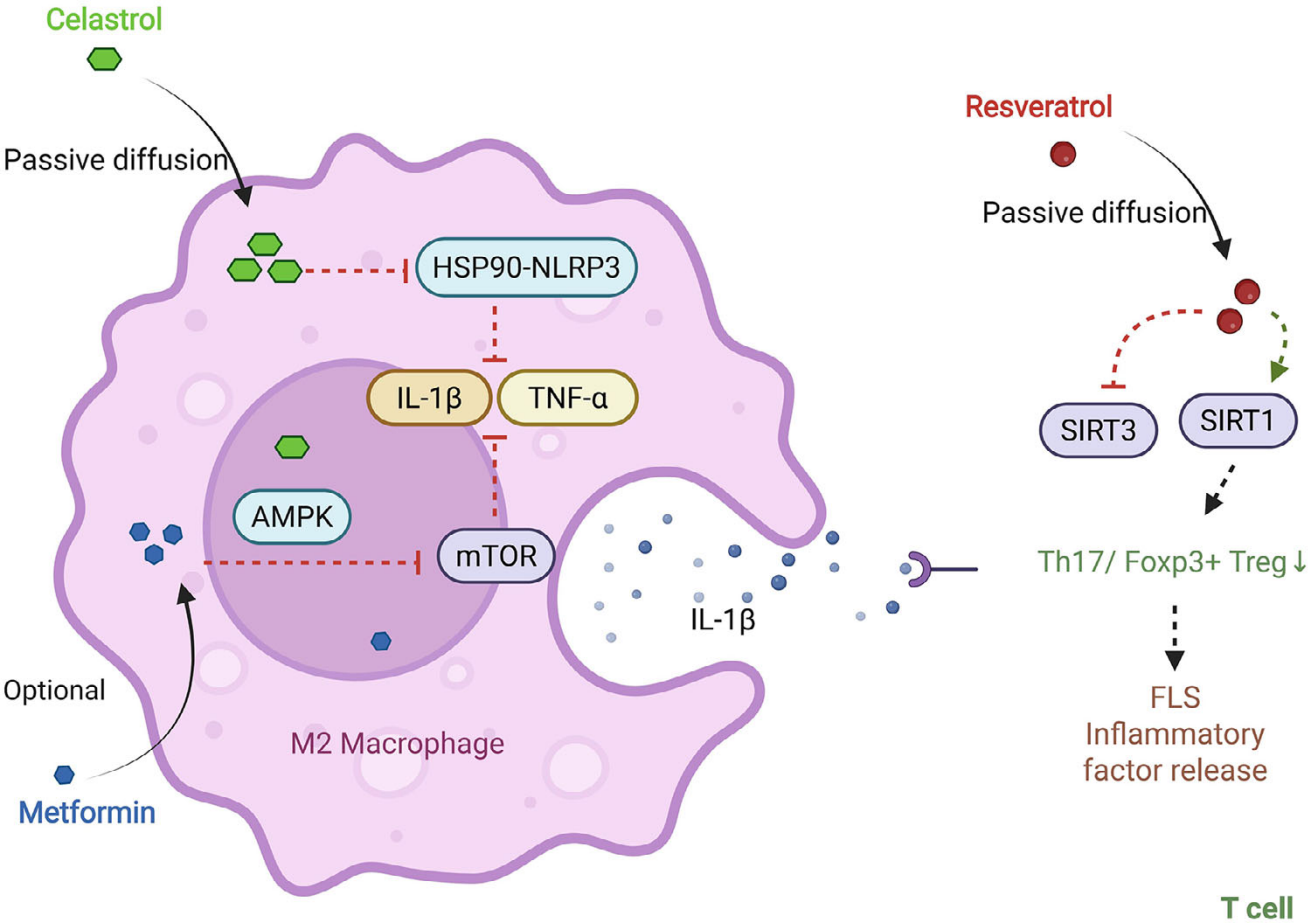
Regulatory mechanisms of small‐molecule drugs on immune cell metabolic lineages. *Note*: AMPK, adenosine 5'‐monophosphate‐activated protein kinase; FLS, fibroblast‐like synoviocytes; Foxp3, forkhead box P3; HSP90, heat shock protein 90; IL‐1β, Interleukin‐1 beta; mTOR, mammalian target of rapamycin; NLRP3, NOD‐like receptor protein 3; SIRT1, Sirtuin 1; SIRT3, sirtuin 3; Th17, T helper 17 cells; TNF‐α, tumor necrosis factor‐alpha; Treg, regulatory T cells.

At the level of synovial immune regulation, the AMPK‐mTOR‐sirtuin (SIRT) signalling axis represents a central target shared by various small molecules (Figure 6) [87]. Metformin activates AMPK signalling to suppress mTOR activation in macrophages, promoting polarisation from the pro‐inflammatory M1 phenotype to the anti‐inflammatory M2 subtype. This shift reduces inflammatory cytokines such as IL‐1β and TNF‐α, thereby alleviating synovial inflammation [88, 89]. Resveratrol further engages the SIRT1‐STAT3 pathway to inhibit Th17 cell differentiation and enhance Treg cell recovery, thereby restoring immune homeostasis within the synovium. It also mitigates Th17‐induced inflammatory responses in FLS [90]. In addition, celastrol suppresses the heat shock protein 90 (HSP90)‐NLRP3 inflammasome axis, thereby blocking IL‐1β activation. Through this mechanism, it indirectly maintains T cell lineage balance and exhibits synergistic immunometabolic regulatory effects [91]. Natural compounds such as curcumin and quercetin can reduce the production of inflammatory mediators by suppressing inflammatory transcriptional programs, including NF‐κB signalling [75, 78]. In addition, bioactive components derived from Tripterygium wilfordii, such as celastrol or triptolide, can exert inhibitory effects at the level of inflammasome activation or cellular stress pathways, thereby providing an additional strategy for interrupting acute inflammatory amplification processes [91]. As illustrated in Figure 6, these mechanisms summarise the key pathways through which small‐molecule drugs remodel the immunometabolic profiles of macrophages and T cells.

#### 3.2.2 Metabolic‐Mitochondrial Axis: Restoring Energy Homeostasis and Reducing Oxidative Stress

**FIGURE 7:**
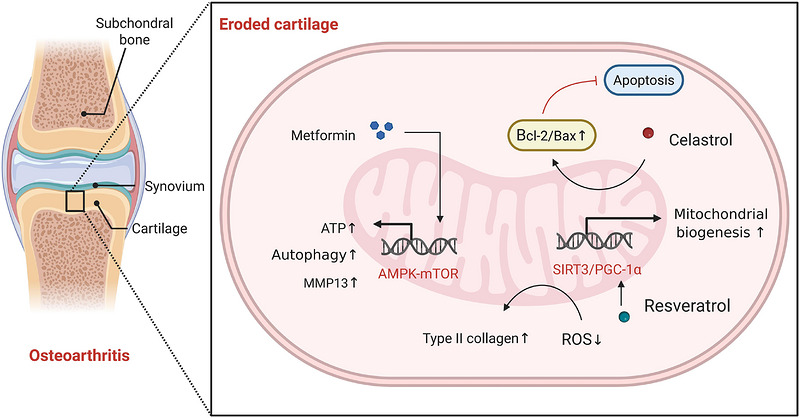
Mechanisms by which small‐molecule drugs modulate mitochondrial function and ROS levels in cartilage. *Note*: AMPK, adenosine 5'‐monophosphate‐activated protein kinase; ATP, adenosine triphosphate; Bax, Bcl‐2‐associated X protein; Bcl‐2, B‐cell lymphoma 2; MMP13, matrix metalloproteinase 13; mTOR, mechanistic target of rapamycin; PGC‐1α, peroxisome proliferator‐activated receptor gamma coactivator 1‐alpha; ROS, reactive oxygen species; SIRT3, sirtuin 3.

At the chondrocyte level, energy homeostasis and mitochondrial functional restoration are central to metabolic reconstruction (Figure 7) [49]. Figure 7 illustrates the principal regulatory axes involved in this process, as well as the major pathways responsible for ROS regulation and control. Studies have shown that in OA chondrocytes, mitochondrial membrane potential is reduced, ATP production capacity is impaired and ROS accumulate excessively, leading to increased expression of matrix‐degrading enzymes such as MMPs and ADAMTS [92]. Metformin activates autophagy through the AMPK‐mTOR signalling pathway and restores intracellular ATP levels. In contrast, resveratrol stimulates the SIRT3/PGC‐1α axis to promote mitochondrial DNA replication and oxidative phosphorylation, thereby reducing ROS accumulation and suppressing MMP‐13 overexpression [68, 93]. Animal studies have demonstrated that both agents increase type II collagen content and improve mitochondrial quantity and morphology, indicating that dual‐axis restoration of metabolism and mitochondrial function constitutes a fundamental mechanism for chondroprotective reversal in OA [75]. In addition, Celastrol has been shown to reduce chondrocyte apoptosis and inflammatory responses by inhibiting endoplasmic reticulum (ER) stress‐related pathways, including ATF6/CHOP, as well as toll‐like receptors‐2/NF‐κB (TLR2/NF‐κB) signalling [94, 95].

#### 3.2.3 Intercellular Communication Axis: Modulating Synovium‐Cartilage Crosstalk and Catabolic Networks

**FIGURE 8:**
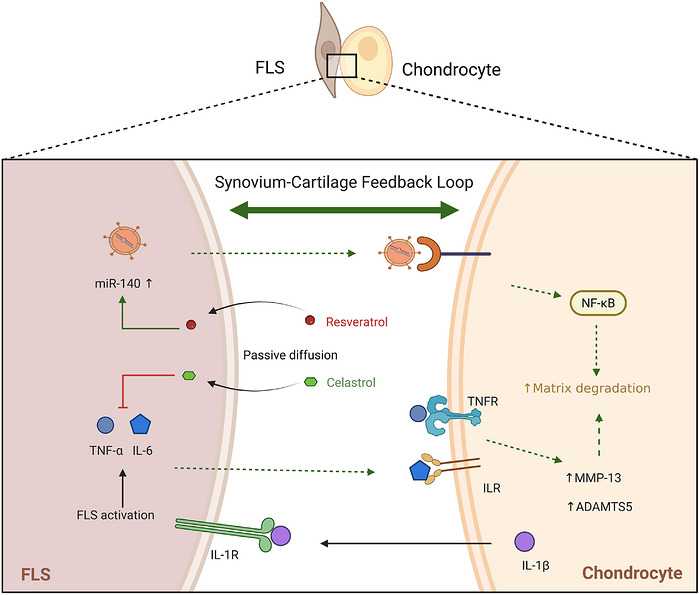
Intercellular signaling interactions between synovial and cartilage cells and the modulatory mechanisms of small‐molecule interventions. *Note*: ADAMTS5, a disintegrin and metalloproteinase with thrombospondin motifs 5; FLS, fibroblast‐like synoviocytes; IL‐6, interleukin‐6; ILR, IL‐1 receptor; MMP‐13, matrix metalloproteinase 13; NF‐κB, nuclear factor kappa B; TNF‐α, tumor necrosis factor‐alpha; TNFR, TNF receptor.

The pathological progression of OA also involves signal crosstalk between the synovium and cartilage (Figure 8, which illustrates the intercellular inflammatory cascade between synovial and cartilage tissues as well as the intervention nodes of small‐molecule agents within this network). Small‐molecule drugs can interrupt the inter‐tissue propagation of inflammatory signals by targeting the NF‐κB and JAK/STAT pathways. Under stimulation with IL‐1β, FLS secrete inflammatory cytokines such as TNF‐α and IL‐6, which subsequently activate cartilage degradation. This effect is significantly attenuated by treatment with resveratrol [96]. Tofacitinib, a JAK/STAT3 inhibitor, reduces synovial cell infiltration and angiogenesis, effectively disrupting the upstream amplification of inflammatory cascades [97]. In parallel, resveratrol and celastrol reprogram intercellular signalling by modulating exosomal microRNA composition—specifically by downregulating miR‐155 and upregulating miR‐140—thereby reducing NF‐κB activation and matrix degradation [98]. Metabolic modulators such as metformin and 5‐aminosalicylic acid (5‐ASA) have demonstrated dual potential in anti‐inflammatory regulation and mitochondrial restoration, suggesting their capacity to achieve more integrated therapeutic effects at the synovium‐cartilage interface. Table 3 systematically summarises the multitarget intervention potential of representative small molecules at the synovium‐cartilage interface [99, 100, 101, 102, 103, 104, 105, 106, 107, 108, 109].

**TABLE 3:** Comparison of typical small‐molecule drugs: targets‐mechanisms‐limitations.

Drug Name (PMID)	Category	Target/mechanism	Clinical stage	Main findings and advantages	Limitations or issues
Lorecivivint (SM04690) [99]	Others (Wnt pathway inhibitor)	Wnt/β‐catenin pathway inhibitor	Phase IIb	Relieves pain, improves function, in multiple Phase IIb trials	Wnt pathway regulates multiple tissue homeostasis; inhibitors may cause off‐target effects; structural improvement not yet confirmed in large trials
MIV‐711 [100]	ECM degradation inhibitor	Cathepsin K inhibitor	Phase IIa	Reduces cartilage thinning and osteophyte formation, convenient oral administration	Cathepsin K is also expressed in bone; long‐term inhibition may affect bone remodeling; limited effect on pain relief
5‐Aminosalicylic acid (5‐ASA) [101]	Energy metabolism regulator	Activates PPARγ, modulates OSCAR pathway	Animal studies	Anti‐inflammatory, promotes cartilage regeneration, slows OA progression	Lacks human clinical data; PPARγ activation may induce metabolic side effects; safety profile remains unclear
GLPG1972 [102]	ECM degradation inhibitor	ADAMTS‐5 inhibitor	Phase II	Structural improvement potential, targets protease activity	Inconsistent clinical efficacy; some patients show no significant structural benefit; low responder rate
Sprifermin (rhFGF18) [103]	Cartilage regeneration promoter	Promotes cartilage formation, activates FGFR3 pathway	Phase II	Increases cartilage thickness, improves joint function	High‐dose FGF18 may cause abnormal cartilage overgrowth; long‐term safety and optimal dosing window remain unclear
Metformin [104]	Energy metabolism regulator	Activates AMPK pathway, anti‐inflammatory, antioxidant	Clinical observation	Reduces chondrocyte apoptosis, improves metabolic syndrome‐related OA	AMPK is a key systemic metabolic regulator; activation may lead to unintended metabolic effects; target specificity needs verification
TPCA‐1 [105]	Inflammatory signal inhibitor	IKKβ inhibitor, blocks NF‐κB pathway	Animal studies	Suppresses inflammatory response, protects cartilage	Lacks clinical research support; IKKβ inhibition may impair normal immune responses, causing side effects
Tofacitinib [106]	Inflammatory signal inhibitor	JAK inhibitor, modulates immune inflammation	Approved	Reduces joint inflammation, improves function	Broad‐spectrum JAK inhibition may increase infection risk and cause hematologic abnormalities; safety monitoring required
STX‐0119 [107]	Energy metabolism regulator	STAT3 inhibitor, modulates STAT3/PPARγ pathway	Animal studies	Slows OA progression, suppresses inflammation, promotes cartilage synthesis	STAT3 broadly regulates cellular functions; systemic inhibition may cause cytotoxicity and insufficient target selectivity
Bergenin [108]	Inflammatory signal inhibitor	Inhibits STAT3, NF‐κB and Jun pathways	Animal studies	Suppresses chondrocyte apoptosis and matrix degradation, slows OA progression	Limited to animal studies; lacks systematic pharmacokinetics/toxicology; clinical translation uncertain
Xanthatin [109]	Inflammatory signal inhibitor	Targets STAT3 (Tyr705) inhibition, via NF‐κB pathway	Animal studies	Slows OA progression, suppresses inflammation	Natural product; oral bioavailability and metabolic characteristics unclear; long‐term toxicity needs validation

Overall, small‐molecule drugs exert multidimensional therapeutic effects—including immune modulation, metabolic rebalancing and structural repair—via key signalling axes such as the AMPK‐SIRT metabolic pathway, the SIRT3‐PGC‐1α mitochondrial axis and the NF‐κB/JAK‐STAT inflammatory cascade. These mechanisms provide an integrated theoretical foundation for the systemic treatment of OA and support multi‐target design strategies in SRNSs. Currently, many small‐molecule candidates have progressed to preclinical and clinical evaluation stages. Compounds such as lorecivivint, MIV‐711 and GLPG1972 primarily act by inhibiting the Wnt or ADAMTS pathways to slow cartilage degradation. Meanwhile, metabolic modulators like metformin and 5‐aminosalicylic acid (5‐ASA) have demonstrated promising anti‐inflammatory and mitochondrial‐restorative properties. However, challenges remain regarding consistency of therapeutic efficacy, systemic side effects and long‐term safety (Table 3). Integrating mechanistic insights with translational research findings may offer a stronger foundation for future SRNSs design and personalised treatment strategies.

### 3.3 Barriers to Small‐Molecule Delivery and Smart Responsive Strategies

Despite the notable anti‐inflammatory and metabolic regulatory effects of various small‐molecule drugs in cellular and animal models, their clinical application remains limited by complex pharmacokinetics and delivery challenges. The enclosed structure of the joint cavity and the high vascular permeability of the synovium jointly hinder effective local drug retention. Concurrently, cartilage, as a poorly vascularised tissue with a dense ECM, further restricts drug diffusion and deposition [110, 111]. Most small molecules, such as resveratrol and celastrol, are hydrophobic, exhibit poor water solubility and tend to bind to synovial proteins, significantly reducing the concentration of free drug in the joint space [112]. Additionally, esterases and hepatic enzymes present in synovial fluid and surrounding tissues accelerate drug metabolism, shorten half‐life and make it difficult to maintain a stable and effective local concentration [113, 114].

Furthermore, small‐molecule drugs often exhibit dose‐dependent toxicity. For example, celastrol has a narrow therapeutic window and systemic administration is prone to inducing hepatic and renal toxicity [112]. While stable therapeutic effects can be achieved in animal models through frequent intra‐articular injections, such regimens in human patients increase the risk of adverse reactions and compromise medication adherence [115]. Moreover, OA lesions demonstrate significant spatial and temporal heterogeneity; pathological variations across different regions of the synovium and cartilage limit the efficacy of a single drug delivery strategy in achieving uniform intra‐articular distribution [116]. These factors collectively make it difficult to strike a balance between sustained local efficacy and systemic safety in the application of small‐molecule therapies.

To address these challenges, SRNSs have emerged as a promising translational strategy for small‐molecule therapeutics. OA lesions are typically characterised by pathological features such as acidic pH, excessive ROS and upregulation of MMPs, which can serve as endogenous triggers for drug release. By incorporating acid‐sensitive, ROS‐responsive and enzyme‐sensitive elements, smart delivery systems enable selective drug release within the lesion microenvironment, achieving precise control through a ‘pathological recognition‐on‐demand release‐sustained action’ mechanism. These systems not only enhance drug accumulation and retention in synovial and cartilage regions but also significantly reduce systemic toxicity and nonspecific distribution. Consequently, smart delivery strategies offer a viable approach to overcoming the limitations of conventional small‐molecule therapies, particularly in terms of stability and bioavailability, while also laying a theoretical foundation for the future optimisation of carrier structures and lesion‐specific design.

## 4 Structure Design and Microenvironmental Adaptation Mechanisms of SRNSs

### 4.1 Pathological Signals in the OA Microenvironment and Principles of Responsive Strategies

**FIGURE 9:**
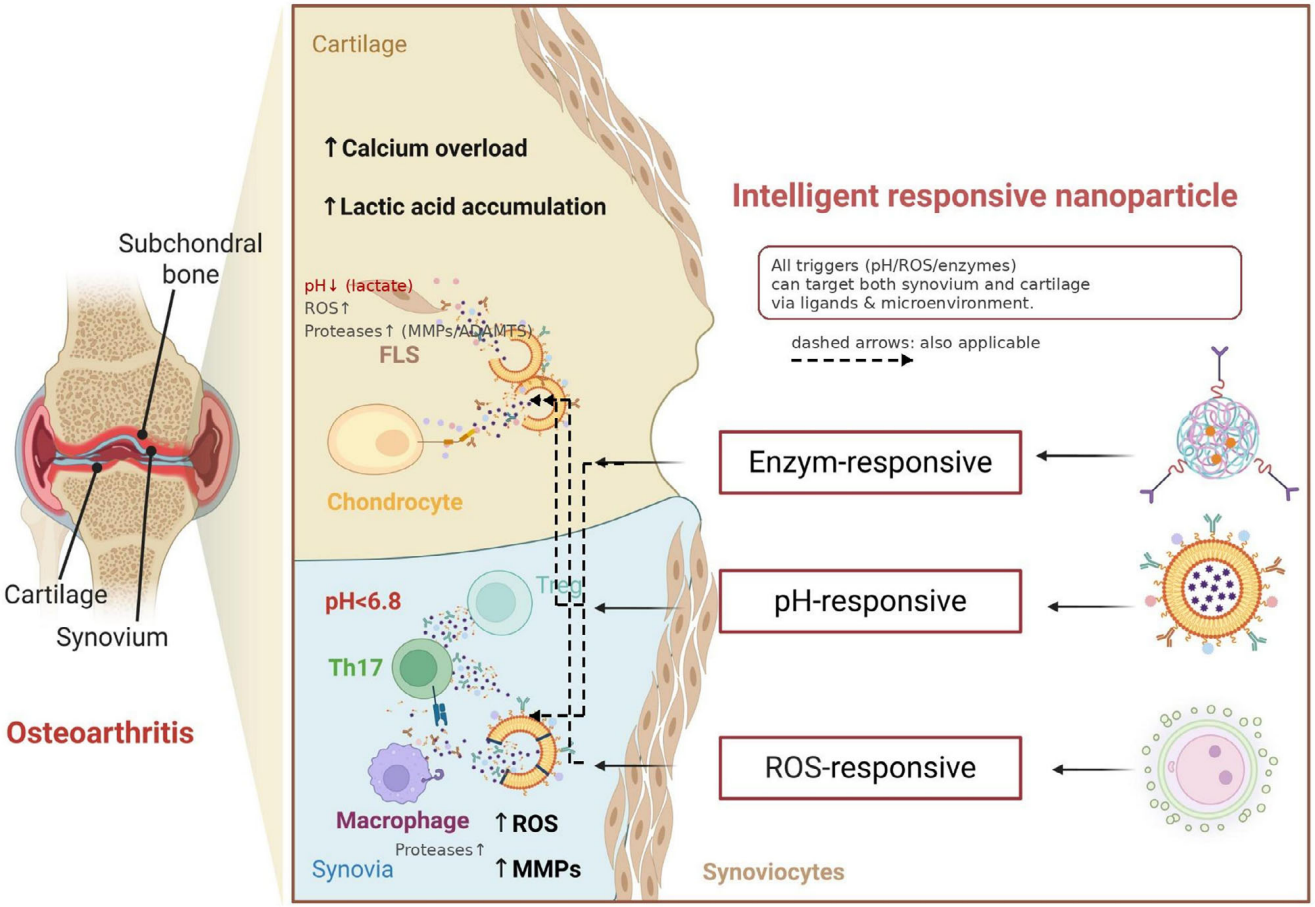
Cross‐adaptive mechanisms between synovium–cartilage microenvironmental features (acidic pH/ROS/enzymes) and stimuli‐responsive delivery systems. *Note*: Solid arrows indicate the most representative stimulus–response pathways in each pathological region, whereas dashed arrows highlight that mild acidity, elevated reactive oxygen species (ROS) and increased expression of matrix‐degrading enzymes are in fact broadly intertwined throughout the OA joint microenvironment. ADAMT, a disintegrin and metalloproteinase with thrombospondin motifs; FLS, fibroblast‐like synoviocyte; MMPs, matrix metalloproteinases; ROS, reactive oxygen species; Th17, T helper 17 cell; Treg, regulatory T cell.

OA lesions are characterised by distinct inflammatory and metabolic abnormalities. The synovium, cartilage and synovial fluid together form a highly dynamic pathological microenvironment that offers intrinsic ‘trigger signals’ for SRNSs. Multiple studies have indicated that the synovial microenvironment in OA joints tends to be mildly acidic, primarily due to enhanced glycolysis in synovial cells and subsequent lactate accumulation [117]. Concurrently, elevated levels of ROS have been observed in OA lesions, with sustained generation of superoxide anions (O_2_^−^) and hydroxyl radicals [118]. This oxidative stress environment not only activates the NF‐κB signalling pathway but also exacerbates chondrocyte apoptosis and ECM degradation. In addition, the expression of various proteolytic enzymes—including MMPs (MMP‐2 and MMP‐13) and esterases—is significantly upregulated in both synovial and cartilage tissues. These enzymes contribute to sustained matrix breakdown and the amplification of inflammatory responses. Collectively, these biochemical aberrations constitute the core features of the OA microenvironment and provide exploitable chemical cues for designing stimuli‐responsive drug release mechanisms in nanocarrier systems (Figure 9).

pH‐responsive systems operate by incorporating protonatable or acid‐sensitive moieties—such as poly(β‐amino ester) (PBAE) and acrylic acid derivatives—into the nanocarrier structure. Upon entering an acidic microenvironment, these polymer chains undergo protonation or structural disassembly, thereby triggering the release of the encapsulated drug [119]. Such systems exhibit high sensitivity in the inflammatory synovial milieu, enhancing cellular uptake by synovial cells and improving overall drug bioavailability. Metal‐organic frameworks (MOFs), such as ZIF‐8, also exhibit acid‐degradable properties and can rapidly release their payload in response to local pH reduction. However, the release rate and concentration of Zn^2+^ must be carefully regulated to avoid potential cytotoxicity [120].

ROS‐responsive systems are designed to trigger drug release in response to elevated levels of H_2_O_2_ or O_2_^−^ within pathological sites. These systems typically rely on oxidation‐sensitive chemical linkages—such as thioethers, thioketals, selenoethers and aryl boronic acids (or esters)—that undergo cleavage upon exposure to ROS‐rich environments. This cleavage leads to structural collapse of the carrier and subsequent drug release [121, 122]. Such strategies have been applied in OA for ROS‐triggered delivery of drugs like dexamethasone, demonstrating the ability to suppress NF‐κB signalling and alleviate cartilage damage [118].

Enzyme‐responsive systems are designed by incorporating peptide sequences or ester bonds that are selectively cleaved by MMPs or esterases. These systems achieve localised degradation and controlled drug release at lesion sites. For instance, collagenase‐sensitive hydrogels and MMP‐13‐responsive polymer platforms have been applied for dual modulation of synovium and cartilage [123]. Recent findings also indicate that Collagenase II and acid phosphatase are markedly upregulated in OA, providing viable enzymatic triggers for responsive hydrogel or carrier design. In one study, a collagenase‐degradable polymer was employed for co‐delivery of TGF‐β and NSAIDs, achieving synchronised regulation of both synovial and cartilage tissues [124, 125].

With advances in research, multi‐responsive mechanisms have gained increasing attention. Systems coupling pH‐ROS or ROS‐enzyme responses enable hierarchical activation and spatiotemporal drug release in complex microenvironments, significantly enhancing delivery specificity and stability. For example, pH/ROS dual‐responsive nanogels have demonstrated stepwise activation in the OA microenvironment—initial structural loosening induced by acidity is followed by ROS‐mediated shell degradation, facilitating deep drug release [126]. Similarly, enzyme/ROS co‐responsive platforms leverage MMP‐triggered release of ROS scavengers; hybrid systems combining selenized polymers with peptide chains have shown remarkable efficacy in delaying cartilage degeneration in the DMM model [123]. Various response combinations offer distinct advantages in targeting, release timing and carrier stability, indicating that intelligent delivery materials can support more refined OA intervention strategies [127].

In recent years, several studies have systematically summarised the correspondence between abnormalities in the OA microenvironment and stimuli‐responsive materials. Notably, to overcome the limitation that single nanocarriers are rapidly cleared from the joint cavity by synovial fluid, current intelligent delivery systems frequently adopt a ‘nano‐macro’ hybrid design. As shown in Table 4 [121, 122, 128, 129, 130], stimuli‐responsive nanoparticles are embedded within scaffolds such as hydrogels or nanofibrous membranes. This strategy not only preserves the sensitive responsiveness to pH, ROS and enzymatic cues, but also significantly prolongs local drug retention, thereby enabling more sustained and tissue‐specific drug delivery. These findings suggest that the chemical characteristics of OA lesions serve not only as pathological markers but also as functional cues for precision therapy. Smart responsive strategies, by detecting and converting these pathological signals, enable a delivery paradigm of ‘disease‐triggered, on‐demand and sustained local release,’ thereby achieving a balance between therapeutic efficacy and safety in anti‐inflammatory and metabolic regulation. Moving forward, the choice of responsive mechanisms should be co‐designed with the drug's physicochemical properties and the carrier's structural characteristics, forming an integrated delivery system that offers more engineering‐driven, translational solutions for targeted arthritis therapy.

**TABLE 4:** Summary of OA microenvironmental abnormalities and the corresponding application of smart hierarchical delivery systems.

Microenvironment abnormality	Response type	Material example	Delivered drug	Target mechanism	Animal model	Therapeutic effect
Acidic (pH ∼6.5) [128]	pH‐responsive	PCL/PEG‐Naringin nanofiber membrane	Naringin	Antioxidant, anti‐inflammatory	DMM mice	Promotes cartilage regeneration, suppresses synovitis
ROS‐enriched [121]	ROS‐responsive	siRNA‐Fe_3_O_4_ nanoparticles in PBA‐modified HA hydrogel	siRNA	mRNA interference, suppresses inflammatory factors	DMM mice	Alleviates synovitis and cartilage damage
Enzyme overexpression (MMP‐13) [129]	Enzyme‐responsive	MMP‐13‐sensitive peptide‐crosslinked HA‐MA hydrogel microspheres (HAM‐SA@HCQ)	Hydroxychloroquine	Inhibits MMPs, alleviates cartilage degradation	DMM mice	Cartilage protection, inflammation relief
Acidic + ROS [122]	Dual‐responsive	ZIF‐8@Quercetin nanoparticles embedded in hydrogel	Quercetin	ROS scavenging, NF‐κB suppression	DMM mice	High delivery efficiency, structural integrity
Enzyme + ROS [130]	Multi‐responsive	MMP‐sensitive peptide + selenized polymer/ROS‐responsive structure	ROS scavenger (e.g., Celastrol)	ROS elimination, targeted synovium/cartilage protection	DMM mice	Effectively suppresses inflammation and ROS oxidative damage

### 4.2 Principles of Synergy between Structural Types and Responsive Design

In OA therapy, the structural characteristics of nanodelivery systems directly influence drug encapsulation efficiency, responsiveness to stimuli and tissue‐targeting capacity. By aligning material properties with pathological microenvironmental signals such as pH, ROS, or enzymes, various systems have been engineered to form a multilayered ‘structure‐response‐effect’ delivery network [117]. Currently, the most extensively studied carrier types include polymeric nanoparticles, MOFs, nanogels and lipid‐polymer hybrid systems.

Polymeric nanostructures represent the most commonly used smart carrier platforms, with PLGA, polyethene glycol (PEG) and their copolymers serving as prototypical examples. These materials exhibit favourable biocompatibility and tunable biodegradability and are frequently combined with pH‐ or ROS‐responsive moieties to enable controlled release through an ‘outer‐layer protection‐core release’ design. For instance, in PLGA‐PEG core‐shell systems, the incorporation of disulfide crosslinking networks allows for cleavage under elevated H2O2 levels, triggering the release of small molecules such as Metformin or Curcumin, thereby achieving a dual function of antioxidation and metabolic regulation [131, 132].

MOFs, such as ZIF‐8, offer high specific surface areas and reversible metal‐ligand coordination, making them ideal platforms for pH/ROS dual‐responsive delivery. These frameworks disintegrate under acidic conditions to release encapsulated drugs, while under ROS‐enriched environments, further breakdown releases Zn^2+^ ions that enhance anti‐inflammatory responses [129]. As a result, ZIF‐8 has been widely applied in delivering polyphenolic or terpenoid natural compounds—such as Celastrol and Quercetin—to achieve synergistic effects in inflammation suppression and ROS scavenging.

**FIGURE 10:**
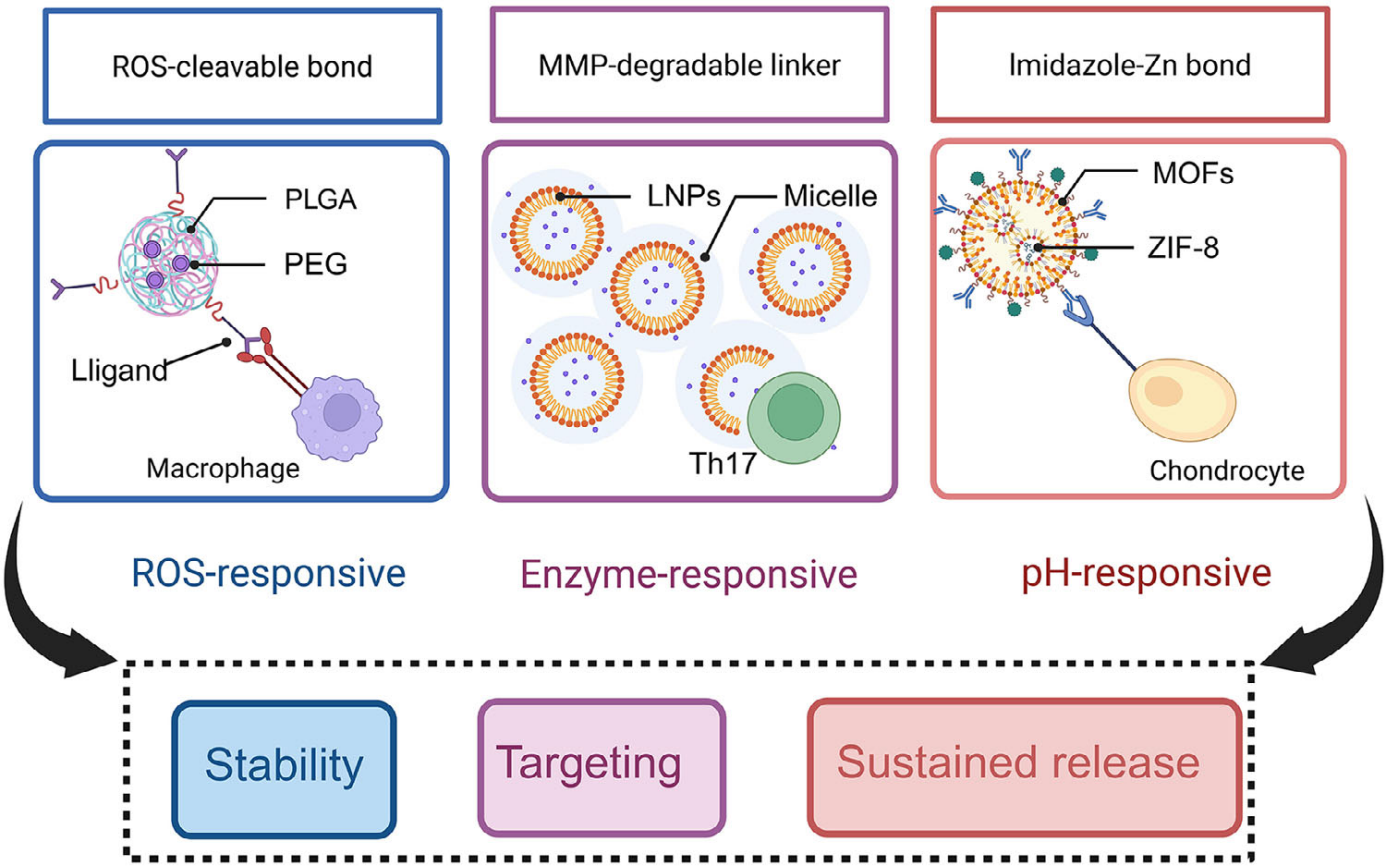
Structural categories and release pathways of SRNSs. *Note*: This diagram presents representative basic types. LNPs: lipid nanoparticles; MMP, matrix metalloproteinase; MOFs, metal‐organic frameworks; PEG, polyethylene glycol; PLGA, poly(lactic‐co‐glycolic acid); ROS, reactive oxygen species; ZIF‐8, zeolitic imidazolate framework‐8.

Nanogel structures possess favourable tissue affinity and a hydrophilic network, enabling gradual degradation in enzyme‐rich environments and thereby supporting sustained drug release. By incorporating MMP‐ or esterase‐cleavable linkages into the gel network, spatially specific release can be achieved in regions of cartilage degradation. Recent studies have further integrated pH‐ and enzyme‐responsive mechanisms, allowing the gel to rapidly disassemble in the acidified synovial environment while continuing controlled release in MMP‐overexpressing areas, thereby enabling cross‐tissue dynamic therapy (Figure 10) [132, 133].

Hybrid systems, on the other hand, combine the advantages of diverse materials to construct multilayered responsive platforms, such as lipid‐polymer composite nanoparticles, cell membrane‐coated nanocarriers and self‐assembling nucleic acid structures. Lipid‐polymer hybrids integrate the flexible encapsulation capacity of liposomes with the structural stability of polymers, making them suitable for delivering hydrophobic small molecules or RNA‐based therapeutics [131]. Cell membrane camouflage enhances immune evasion and synovial targeting, while self‐assembling nucleic acid nanoparticles—with their programmable configurations and degradability—have been employed to interfere with MMP or TNF signalling pathways, effectively suppressing inflammatory cascades [134].

Overall, an ideal SRNS for OA treatment should exhibit three core features: structural stability, stimulus responsiveness and tissue penetrability. Structural stability ensures adequate drug loading and prolonged in vivo circulation time; stimulus responsiveness enables on‐demand drug release in the pathological microenvironment; and tissue penetrability determines whether the drug can traverse synovial barriers and cartilage matrices to exert precise therapeutic effects (Figure 10). By optimising the interplay between material properties and pathological signals, it is possible to achieve a systematic design of drug delivery systems, thereby offering more engineered and translational solutions for precision OA therapy.

### 4.3 Multi‐Targeting Mechanisms and Selectivity Enhancement Strategies

The therapeutic efficacy of smart nanodelivery systems depends largely on their ability to precisely target key pathological tissues such as the synovium and cartilage [135].

Synovial targeting strategies primarily exploit the recognition of cell surface receptors or local electrostatic differences to achieve specific accumulation. Among these, hyaluronic acid (HA)‐modified systems represent the most prominent synovial targeting approach. HA binds to cluster of differentiation 44 (CD44) receptors expressed on FLS and macrophages, facilitating receptor‐mediated endocytosis. This mechanism not only enhances synovial tissue accumulation but also significantly prolongs intra‐articular drug retention [136]. Another critical targeting strategy involves the specific recognition of immune cells. By decorating nanocarriers with ligands for CD86 or CCR6, it is possible to selectively identify pro‐inflammatory M1 macrophages and Th17 cells, thereby enabling precise modulation of inflammatory cell populations [137]. For example, in a study involving a synovial macrophage‐targeted delivery system, anti‐inflammatory drugs were administered via carriers modified with macrophage receptor ligands, resulting in a significant reduction in joint inflammation [138]. Additionally, pH‐responsive charge‐reversal mechanisms offer an effective means of enhancing cellular uptake. Polymers modified with PDEA, PEI, or histidine (His) can undergo a charge switch from negative to positive when the pH falls below 6.8, thereby increasing electrostatic interactions with synovial cell membranes and promoting endocytosis [135]. Collectively, these multilayered synovial targeting strategies significantly improve the specificity and local concentration of drug delivery, while also mitigating systemic toxicity and off‐target effects.

In contrast, cartilage‐targeted delivery focuses primarily on overcoming matrix barriers and enhancing local drug deposition. One of the main strategies involves targeting the ECM by recognising type II collagen or cartilage‐specific proteins to promote drug accumulation within the cartilage layer. For example, type II collagen‐binding peptides and RGD peptides are commonly employed to develop nanosystems capable of penetrating the cartilage matrix [139]. These peptide sequences selectively bind to the collagen triple helix or integrin receptors, enabling targeted delivery to chondrocytes and sustained drug release, thereby facilitating matrix synthesis and tissue regeneration. Additionally, certain systems incorporate polycationic modifications or nanogel‐based sustained‐release networks to prolong drug retention in the deep cartilage zone, allowing for extended local delivery of anti‐inflammatory and antioxidant agents.

**FIGURE 11:**
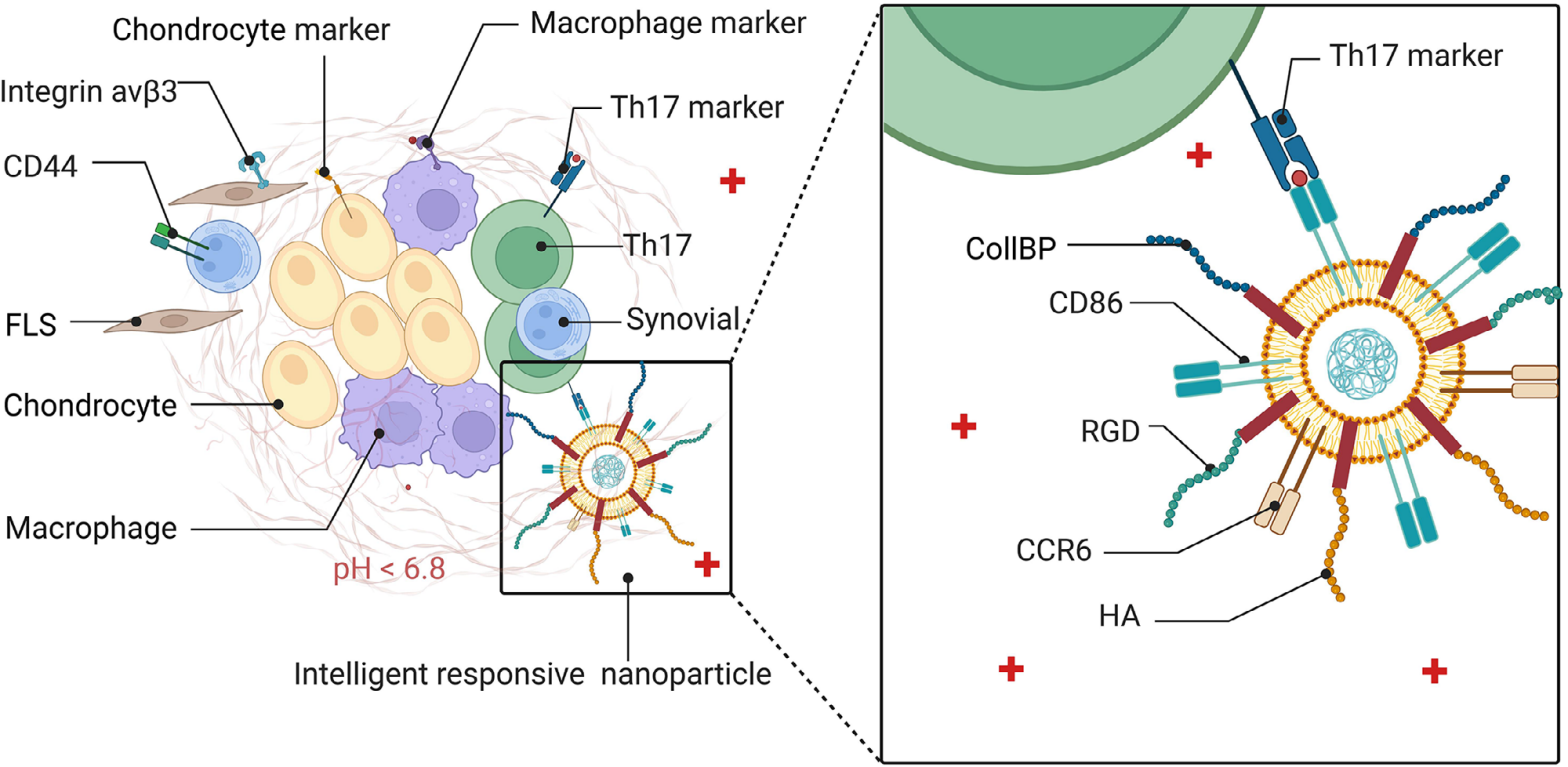
Targeted delivery strategies and surface modification mechanisms for synovium and cartilage. *Note*: CCR6, C‐C chemokine receptor type 6; CD86, cluster of differentiation 86, A co‐stimulatory molecule; CollBP, collagen‐binding protein; HA, hyaluronic acid; FLS, fibroblast‐like synoviocyte; RGD, arginine‐glycine‐aspartic acid sequence; Th17, T‐helper 17 cells; Th17 marker, marker of T‐helper 17 cells.

Multi‐targeting systems integrate both ‘cell‐specific recognition’ and ‘matrix‐binding enrichment’ mechanisms to establish a hierarchical synovium‐cartilage delivery axis (Figure 11). This approach operates via a coordinated strategy: targeting synovial inflammatory cells in the outer layer and penetrating the metabolically active cartilage region in the inner layer. Such systems enable a spatially resolved delivery process characterised by lesion recognition, tissue penetration and sustained release [140]. This multidimensional targeting paradigm allows simultaneous modulation of macrophages and T cells in the synovium, as well as metabolic pathways in chondrocytes. It establishes a cross‐tissue and cross‐cellular therapeutic network, offering a robust framework for precision intervention in OA.

When multiple targeting ligands are conjugated onto a single nanocarrier, the central engineering challenge is to balance multi‐tissue/multi‐cell targeting efficiency with physicochemical and biological constraints, including particle size, surface charge, immunocompatibility and avoidance of ligand interference. To reduce potential conflicts between synovial targeting and cartilage targeting, a more feasible strategy is hierarchical delivery or staged exposure. In this approach, nanoparticles first achieve synovial enrichment, followed by microenvironment‐triggered exposure of a secondary targeting module—activated by stimuli such as pH, ROS, or specific enzymes—to enhance cartilage deposition. However, multiligand conjugation may lead to several complications, including increased particle size, altered surface charge, enhanced protein corona formation and complement activation and reduced binding efficiency due to steric hindrance among ligands. Therefore, it is important to prioritise optimisation of ligand density and ratio and to employ spacer arms to achieve spatial separation between ligands. When necessary, stealth coatings can be incorporated to reduce nonspecific adsorption. For validation, it is essential to compare single‐targeting versus multitargeting systems in terms of cellular uptake by macrophages, FLS and chondrocytes, as well as ex vivo cartilage penetration and intra‐articular distribution, to determine whether synergistic effects or trade‐offs occur. If significant interference between ligands is observed, an alternative strategy is to revert to combined or sequential administration of two single‐targeting carriers rather than integrating multiple ligands onto a single platform.

### 4.4 Biological Validation and Application Prospects of Smart Responsive Systems

**FIGURE 12:**
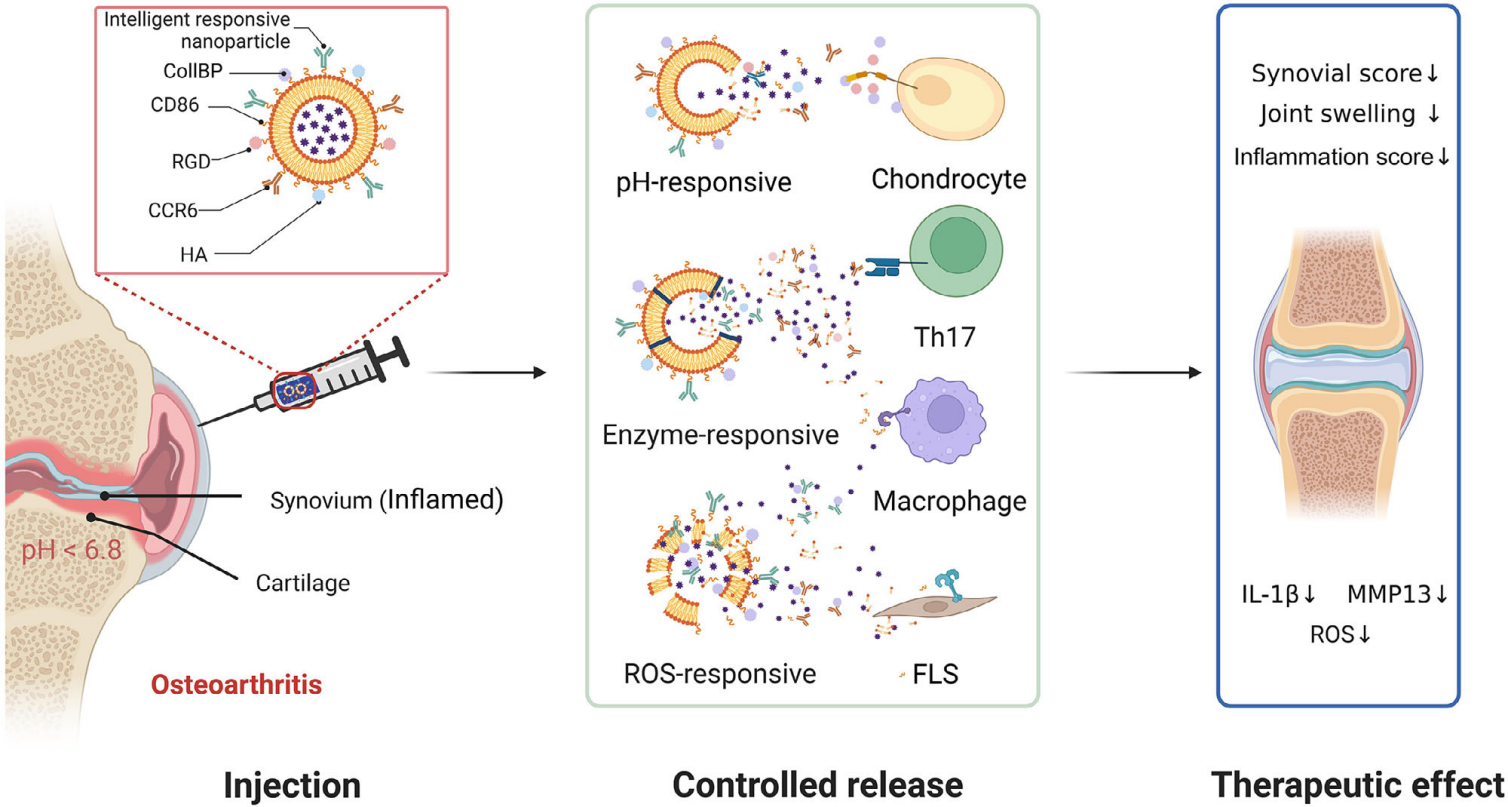
Therapeutic mechanisms and lesion‐targeting pathways of representative SRNSs. *Note*: CCR6, C‐C chemokine receptor type 6; CD86, cluster of differentiation 86; CollIBP, collagen‐binding peptide; FLS, fibroblast‐like synoviocytes; HA, hyaluronic acid; IL‐1β, interleukin‐1 beta; MMP13, matrix metalloproteinase 13; RGD, arginine‐glycine‐aspartic acid; ROS, reactive oxygen species;Th17, T‐helper 17 cells.

In recent years, SRNSs designed based on the pathological signalling features of the OA synovium‐cartilage microenvironment have been systematically validated in animal models (Figure 12). Multiple studies have demonstrated that these systems can achieve localised drug activation and sustained release within acidic and ROS‐enriched lesion environments, thereby markedly enhancing the therapeutic efficacy of small‐molecule drugs while reducing systemic toxicity [141, 142]. For instance, a ZIF‐8‐based carrier modified with HA was employed to deliver Celastrol, achieving dual pH/ROS responsiveness. This system significantly suppressed MMP13 expression and promoted Col2a1 synthesis in both IL‐1β‐stimulated FLS and ACLT rabbit models, exhibiting strong CD44‐mediated synovial targeting and cartilage‐penetrating capabilities [136]. Another study incorporated a ROS‐sensitive thioether crosslinked network into a PLGA‐PEG core‐shell nanoparticle, enabling H_2_O_2_‐triggered release of Metformin. This approach effectively inhibited glycolysis in FLS, reduced ROS levels and NF‐κB activation and ultimately alleviated synovial inflammation and joint swelling [143]. In addition, notable progress has been made in immune cell‐targeting strategies. For example, CD86 antibody‐modified liposomes were used to deliver DMF, allowing precise targeting of pro‐inflammatory M1 macrophages. In the DMM model, this system significantly downregulated iNOS and TNF‐α expression while promoting M2 polarisation [144].

These systems have been validated across multiple dimensions in animal models, demonstrating that smart delivery platforms not only improve drug distribution and local concentration but also enable dual modulation of inflammation and metabolism along the synovium‐cartilage axis (Table 5) [106, 107, 108, 109, 132, 145, 146, 147]. Mechanistically, they achieve controlled drug release in response to acidic and oxidative stimuli, thereby modulating macrophage polarisation, suppressing hyperactive metabolism and pro‐inflammatory cytokine secretion by FLS in the synovium and preserving mitochondrial function and matrix synthesis in cartilage [148]. Immunohistochemical and RNA sequencing analyses further revealed that delivery of agents such as resveratrol via ROS‐responsive systems significantly reduced MMP‐13 and ADAMTS5 expression in synovium‐cartilage co‐culture models, thereby disrupting inflammatory signalling crosstalk between these two tissues [149]. Similarly, reversal of the Th17/Treg ratio and restoration of mitochondrial membrane potential corroborated the therapeutic potential of these systems in re‐establishing immune homeostasis and correcting energy metabolism [150]. Taken together, smart responsive systems have established a multilayered therapeutic paradigm in animal studies—characterised by local activation, immune modulation, metabolic remodelling, and tissue regeneration—providing a solid foundation for the precision treatment of OA.

**TABLE 5:** Summary of smart nanodelivery systems.

Material/system	Response mechanism	Loaded substance	Target tissue/cells	Experimental model and findings	Limitations or challenges
PCL/PEG‐NAR nanofiber membrane [107]	pH‐responsive	Naringin	Cartilage	Ester bond cleavage releases naringin in acidic environment, slows OA progression	Limited mechanistic clarity, lacks multi‐pathway validation and preclinical pharmacokinetic support
Rh‐PLGA‐NPs@NH_4_ nanoparticles [108]	pH‐responsive	Rhein	Cartilage	Generates NH_4_, CO_2_, H_2_O in weakly acidic joint cavity, ruptures nanoparticle shell for burst release	Lacks systematic toxicology evaluation and validation of long‐term sustained release effects
HMPBzymes nanoplatform [109]	pH‐responsive	None	Macrophages	Induces M1‐to‐M2 macrophage polarization, slows OA progression	Potential immunogenicity of nanostructures; requires in situ reactivity and tissue compatibility testing
Que‐Mg@SA microspheres [145]	ROS‐responsive	Quercetin‐magnesium complex	Cartilage	Anti‐inflammatory, antioxidant, protects chondrocytes, slows ECM metabolic imbalance	Mainly based on animal models, lacks cross‐species extrapolation and clinical sample validation
PMES‐TO@1@POM nanoplatform [146]	pH‐responsive	Pomalidomide	Cartilage	Releases 79% within 12h at pH 5.8, enhances therapeutic efficacy	Lacks systematic toxicology evaluation and validation of long‐term sustained release effects
HA‐Lipo‐DIC/DEX nanoparticles [106]	pH‐responsive	Diclofenac and Dexamethasone	Cartilage	Prolongs intra‐articular drug release, reduces joint pain	Mainly based on animal models, lacks cross‐species extrapolation and clinical sample validation
Multi‐responsive smart hydrogel [132]	Enzyme/pH/ROS/temperature/light‐responsive	Multiple drugs	Cartilage	Enables controllable drug release, promotes cartilage regeneration, relieves inflammation	Limited mechanistic clarity, lacks multi‐pathway validation and preclinical pharmacokinetic support
Chitosan hydrogel system [147]	pH‐responsive	Multiple drugs	Cartilage	Optimizes drug release efficiency, enhances therapeutic outcomes	Lacks systematic toxicology evaluation and validation of long‐term sustained release effects

Building upon these advances, physical stimuli and multimodal synergistic platforms have further expanded the functional boundaries of SRNSs. Techniques such as photothermal therapy (PTT) and sonodynamic therapy (SDT) activate localised temperature elevation or ROS generation through external stimuli, thereby coordinating drug release and microenvironment modulation with chemical‐responsive mechanisms [132, 151]. For instance, a PTT carrier based on black phosphorus nanosheets, modified with RGD peptides to enhance cartilage targeting, raised the local temperature to approximately 42°C under near‐infrared irradiation, triggering drug release and promoting cartilage repair [152]. In SDT systems, polymer microbubbles generated reactive radicals under ultrasound exposure and induced the collapse of drug‐loaded structures, thereby enabling precise ablation of inflamed synovial tissue and synchronised drug release [153]. In addition, emerging platforms such as ROS‐responsive microcapsules and integrated fluorescence‐magnetic resonance theranostic systems have been developed to enable a closed‐loop therapeutic strategy characterised by ‘controlled drug release‐real‐time imaging‐precise intervention,’ exhibiting excellent performance in synovial inflammation scoring, cartilage repair and consistent drug distribution [118].

**FIGURE 13:**
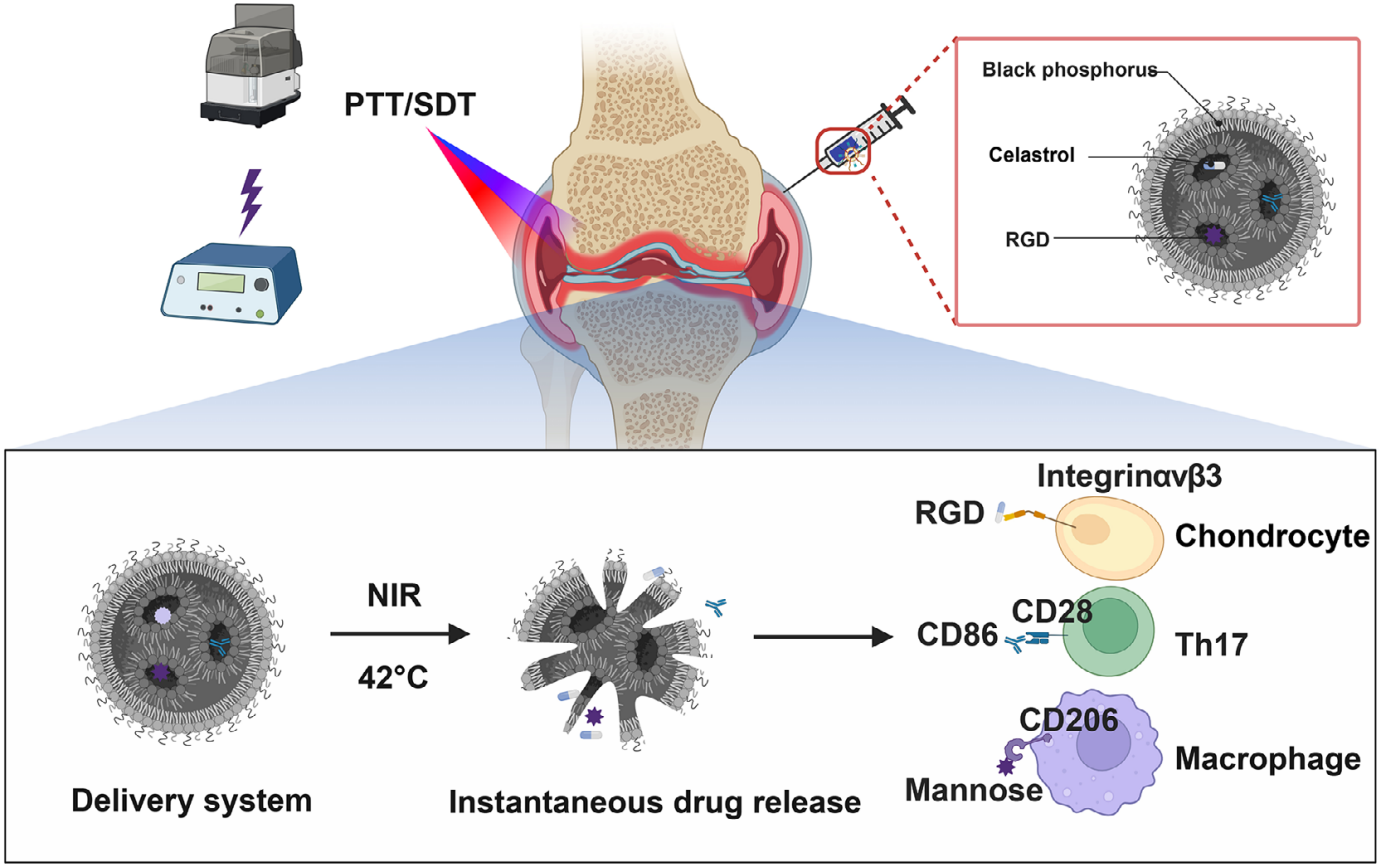
Schematic of synergistic mechanisms in combined therapy systems (nanodelivery + PTT/SDT). *Note*: NIR, near‐infrared light; PTT, photothermal therapy; RGD, arginine‐glycine‐aspartic acid; SDT, sonodynamic therapy; Th17, T helper 17 cells.

Looking ahead, the evolution of SRNSs is expected to shift from single‐drug release platforms toward ‘diagnosis‐therapy integration’ and ‘cell‐responsive’ systems (Figure 13). By combining these systems with stem cell‐derived exosomes, CRISPR‐based gene editing, or immunoregulatory technologies, it becomes possible to achieve visualised, controllable and multi‐tiered drug action. Such intelligent platforms hold great promise for achieving integrated intervention in OA, from inflammation suppression to tissue regeneration, thereby providing new material and engineering solutions for future personalised and programmable OA therapies.

### 4.5 Lubrication‐Enhancing Stimuli‐Responsive Nanodelivery: From Friction Reduction to Functional Restoration

Beyond immunometabolic regulation, the tribological microenvironment of the joint also contributes to structural deterioration in OA. Reduced levels or structural alterations of hyaluronic acid (HA) and proteoglycan 4 (PRG4, lubricin) in synovial fluid weaken cartilage boundary lubrication, thereby increasing contact friction and wear. In vitro tribological studies indicate that HA and PRG4 exhibit synergistic friction‐reducing effects at physiological concentrations and a deficiency of either component compromises boundary lubrication [154]. Consequently, integrated strategies aimed at supplementing or biomimetically reconstructing the lubrication layer while simultaneously delivering anti‐inflammatory and antioxidant agents have gradually emerged as an important branch of intelligent delivery systems.

A representative approach involves constructing a highly hydrated lubricating corona on the nanoparticle surface, such as zwitterionic polymer brushes, to form a stable hydration layer that reduces shear‐induced wear, while therapeutic agents are conjugated through ROS‐ or pH‐sensitive linkages to enable lesion‐triggered drug release. For example, in core‐brush structured nanoparticle platforms, the outer zwitterionic brush layer provides lubrication and anti‐adhesive properties, whereas the inner core loads anti‐inflammatory drugs via ROS‐cleavable linkers. Such systems can simultaneously reduce friction and inflammatory burden within the joint, thereby mitigating cartilage matrix degradation [155].

From an engineering design perspective, lubrication‐enhanced SRNSs must balance the stability of the lubricating layer with the controllability of stimulus‐responsive drug release. This is commonly achieved through a modular architecture, in which the lubricating corona and drug‐loaded core are structurally separated to minimise functional interference. At the same time, careful optimisation of particle size, surface charge and cartilage‐binding capacity is required. By incorporating friction reduction as a quantifiable therapeutic endpoint, this approach expands treatment goals beyond inflammation control toward restoration of joint function, providing a more clinically relevant and system‐oriented strategy for OA therapy.

## 5 Challenges and Future Directions

### 5.1 Safety and Immunogenicity Challenges: Biomimetic and Biodegradable Material Approaches

Although SRNSs have demonstrated outstanding performance in drug delivery efficiency and targeting capability, their long‐term biosafety and immunogenicity remain significant obstacles to clinical translation [156]. Various material sources—such as MOFs, synthetic polymers and liposomes—may produce unpredictable metabolic byproducts in vivo. Some released metal ions can even activate synovial cells, potentially inducing chronic inflammatory responses [157, 158]. Moreover, surface modifications of nanomaterials, including antibodies and PEG, may be recognised by the innate immune system, activating immune responses via toll‐like receptors or the complement pathway, thereby contributing to ‘nanotoxicity’ effects [159].

To address these challenges, biomimetic nanostructures have emerged as a focal point of current research. Coating nanoparticles with macrophage membranes, FLS membranes, or synovium‐derived exosomal membranes can endow the system with ‘self‐recognition’ features, significantly reducing immune recognition and phagocytosis in vivo, thereby achieving an ‘immune stealth’ effect [160]. In addition, the incorporation of biodegradable natural polymers, such as gelatin and HA derivatives, helps avoid the accumulation of toxic residues resulting from prolonged retention [161]. Future work should prioritise comprehensive toxicological assessments across the full material life cycle, including the development of multi‐animal models and long‐term immune monitoring platforms, to facilitate the systematic and forward‐looking establishment of safety evaluation standards.

### 5.2 Integrated Design and Trade‐Offs Between Stimuli Responsiveness and Stealth/Biodegradability

When integrating stimuli responsiveness with stealth and biodegradability, the key principle is structural coupling rather than simple functional stacking. On one hand, stealth layers, such as PEG, zwitterionic polymers, or cell membrane coatings, should ideally be designed as trigger‐responsive outer shells capable of detachment or conformational change. In non‐diseased environments, this outer layer can reduce protein adsorption and complement activation; however, within the OA microenvironment (e.g., acidic pH, elevated ROS, or disease‐associated enzymes), it should be able to shed or transform, thereby exposing the inner responsive release and targeting modules. This strategy helps avoid a scenario in which excessively strong stealth effects shield or suppress the intended stimulus responsiveness. On the other hand, biodegradability should be incorporated into both the structural backbone materials and the stimuli‐sensitive linkages, allowing triggered drug release and material degradation/clearance to occur synchronously. Such a design can reduce the risk of long‐term accumulation, including potential concerns related to residual nanomaterials, metal ion release, or degradation byproduct buildup.

However, this integration inevitably introduces several design trade‐offs. A more stable stealth layer generally improves immunocompatibility, but may simultaneously reduce trigger sensitivity. Additional coatings and multistep surface modifications may increase particle size and alter surface charge, thereby affecting cartilage penetration and intra‐articular pharmacokinetics. While biomimetic materials can effectively reduce immune recognition, they also impose higher requirements regarding source control and batch‐to‐batch consistency. Therefore, the co‐optimisation of responsiveness and safety should rely on a set of core evaluation metrics, including particle size, polydispersity index (PDI) and ζ potential, as well as protein corona formation and complement activation, ion release and metabolic byproduct profiles and in vivo biodistribution and clearance behaviour. These parameters collectively provide a systematic framework for balancing stimulus responsiveness, therapeutic performance and biosafety in advanced nanodelivery systems.

### 5.3 Targeting Efficiency and the Controversy of OA Heterogeneity: Adaptive Boundaries of Smart Delivery Systems

Although SRNSs have demonstrated promising tissue‐targeting capabilities in animal models, their clinical efficacy remains challenged by the heterogeneity of OA lesions. Significant variability in synovial CD44 expression, MMP‐13 levels and ROS concentrations across different stages and patient subtypes of OA has been observed, leading to reduced efficiency of some targeting systems in low‐expression or non‐inflammatory OA phenotypes [162]. For instance, CD44 expression on FLS varies widely among patients, resulting in suboptimal delivery efficiency of HA‐modified carriers in certain individuals [163]. Moreover, the spatial and temporal distribution of pH and ROS levels within the joint cavity is inconsistent, further compromising the precision of stimulus‐responsive delivery systems [164]. These findings raise a central question: can current targeting mechanisms sufficiently overcome OA heterogeneity?

To address the targeting challenges posed by lesion heterogeneity, dynamic alignment between pathological signals and carrier properties must be achieved. scRNA‐seq, spatial transcriptomics and metabolomics have enabled the construction of multidimensional OA subtype maps, distinguishing high‐inflammatory, metabolically dysregulated and ROS‐enriched subtypes [165, 166]. Based on these insights, materials with adaptive microenvironmental responsiveness should be engineered to dynamically adjust surface charge, release thresholds, or cellular uptake pathways under varying inflammatory and metabolic conditions, thereby improving delivery consistency [60]. Furthermore, the development of ‘patient expression profile‐carrier structure’ databases, coupled with artificial intelligence (AI)‐driven predictive and feedback‐adjustment mechanisms, holds the potential to establish truly individualised delivery systems [167].

### 5.4 Integration and Competition between Smart Delivery Systems and Emerging Technologies

Compared with emerging technologies such as gene therapy, cell‐penetrating peptides (CPPs) and stem cell‐derived exosomes, SRNSs exhibit distinct advantages in drug stability and controlled release. However, they remain limited in their ability to provide sustained regulation and facilitate tissue regeneration [141]. Gene therapy enables direct modulation of key pathways such as MMP and HIF‐1α at the transcriptional level, but its clinical application is constrained by off‐target effects and the immunogenic risks associated with viral vectors. CPPs offer excellent membrane‐penetrating capabilities but are susceptible to enzymatic degradation and thus require nanoparticle carriers for stable delivery. Exosomes, due to their intrinsic biocompatibility and tissue affinity, are considered ideal biological vectors, yet challenges in large‐scale production and standardisation persist [168, 169, 170].

Consequently, future efforts should focus on developing hybrid platforms that integrate materials, biologics and genetic elements. For example, co‐loading functional nanomaterials with MSC‐derived exosomes or genetic fragments may achieve simultaneous anti‐inflammatory effects, metabolic remodelling, and cartilage regeneration [171]. These combinatory systems demonstrate unique advantages in bidirectional modulation along the synovium‐cartilage axis, offering a novel paradigm for multi‐level precision interventions.

### 5.5 AI‐Assisted Design and Optimisation of Stimuli‐Responsive Nanodelivery Systems

The value of AI and machine learning (ML) in the development of SRNSs extends beyond candidate drug screening; it also lies in accelerating the predictable design linking formulation and structural parameters to in vivo behaviour. First, AI can learn the relationships between material composition, particle size, surface charge, surface modification and types of responsive linkages and key quality attributes under multi‐objective constraints. Through such modelling, AI can inversely search for optimal parameter combinations that simultaneously satisfy requirements such as joint retention, cartilage penetration, response thresholds and release windows. When integrated with active learning and self‐driving experimental platforms, this approach can substantially reduce trial‐and‐error costs in nanomedicine development [172, 173].

Second, immunogenicity and biosafety can be assessed in advance through modelling of protein corona formation and immune recognition. Previous studies have shown that machine learning models based on the physicochemical characteristics of nanoparticles can predict the functional composition of protein coronas, thereby guiding the selection of surface chemistries and coatings to reduce complement activation and nonspecific phagocytic uptake [174, 175].

Third, for stimuli‐responsive systems, AI can be applied to fit and predict swelling, degradation and drug‐release kinetics under varying conditions of pH, ROS levels, or enzymatic activity. By incorporating physics‐constrained models or physics‐informed neural networks, limited experimental datasets can be extrapolated to a broader condition space. This enables quantitative optimisation of response thresholds and release profile shaping [176].

Fourth, in multitargeted delivery systems, AI can facilitate optimisation of ligand combinations and interfacial presentation. For example, it can perform multi‐objective trade‐off searches among parameters such as synovial uptake, cartilage binding, particle size and immunocompatibility, while identifying the most effective ligand density and ratio ranges. Such approaches help minimise the redundancy and interference risks associated with multiligand conjugation, as discussed in Section 4. Overall, applying AI to inverse design of materials and structures, prediction of immunocompatibility, simulation of stimulus‐responsive drug release kinetics and optimisation of multitargeted interfaces creates a coherent framework aligned with the theme of stimuli‐responsive nanodelivery systems. This integration also provides essential technological support for future personalised delivery strategies and translational nanomedicine engineering.

### 5.6 Future Perspectives

Overall, SRNSs are currently at a critical juncture, transitioning from laboratory validation to clinical translation. Future development must advance in parallel across four key dimensions: safety evaluation, heterogeneity adaptation, technological integration and intelligent evolution.

1. Establish comprehensive biosafety databases to standardise toxicological assessments across species and platforms;
2. Develop OA lesion subtype identification systems based on multi‐omics characteristics to enable personalised targeting;
3. Promote multimodal integration with gene editing, exosome‐based therapies and AI algorithms to construct dynamically controllable therapeutic networks;
4. Explore self‐learning and self‐regulating delivery platforms that endow materials with capabilities for self‐evolution and long‐term adaptability.

**FIGURE 14:**
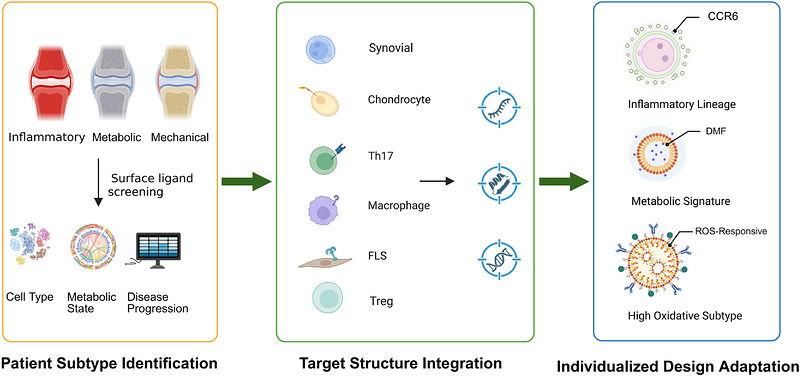
Multi‐omics‐driven OA subtype identification and personalized adaptation of delivery systems. *Note*: CCR6, C‐C chemokine receptor type 6; DMF, dimethyl fumarate; ROS, reactive oxygen species.

Under this emerging paradigm, OA therapy will move beyond simply enhancing drug delivery efficiency, evolving into an integrated intervention framework that combines bioinformatics, materials science and systems medicine. This shift will open new avenues for the precise regulation of chronic degenerative diseases (Figure 14).

## 6 Conclusion

### 6.1 Summary of Current Research Progress and Systematic Integration

OA, a chronic degenerative disease characterised primarily by an imbalance of the synovium‐cartilage axis, has increasingly been recognised as a systemic condition driven by immunometabolic dysregulation, extending beyond the traditional ‘mechanical wear and tear’ paradigm. Based on this updated understanding, anti‐inflammatory small molecules have emerged as critical therapeutic agents capable of reshaping local inflammatory and metabolic environments to delay OA progression. However, conventional drugs are often limited by poor in vivo stability, inadequate tissue penetration and high systemic toxicity, thereby failing to achieve sustained and precise therapeutic effects.

SRNSs have offered a transformative solution for the targeted delivery of small‐molecule drugs. This review begins by examining the mechanisms underlying immunometabolic dysfunction along the synovium‐cartilage axis and provides a comprehensive overview of the classification, mechanisms of action and therapeutic potential of anti‐inflammatory small molecules. It further explores the design principles and structural integration strategies of SRNSs under pathological microenvironmental conditions such as altered pH, elevated ROS and enzyme activity. Additionally, representative research cases, animal‐model validation, combination therapy platforms and supporting figures and tables are reviewed to illustrate systematic advances in this field. Overall, the interplay among mechanistic insights, material innovation and model validation has established a synergistic framework, laying a solid foundation for advancing from functional verification to an integrated ‘structure‐mechanism‐efficacy’ design paradigm. At the same time, an increasing number of studies are incorporating joint lubrication and reconstruction of the tribological microenvironment into the functional objectives of SRNSs. By coupling biomimetic lubricating coronas with stimuli‐responsive drug release, these systems aim to achieve a synergistic ‘friction reduction plus anti‐inflammatory’ effect, thereby more directly promoting restoration of joint function.

### 6.2 Emphasising the Unique Potential of SRNSs in Immunometabolic Regulation of OA

Compared with conventional delivery approaches, SRNSs not only enable high drug‐loading capacity, controlled release and targeted delivery, but also exhibit unique adaptability and integration potential in OA—a disease marked by pronounced spatial heterogeneity and dynamic lesion evolution. Particularly within the context of synovium‐cartilage axis immunometabolic disorders, SRNSs can be precisely activated in response to pathological microenvironmental cues such as acidic pH, ROS accumulation and aberrant MMP expression, thereby maximising therapeutic efficacy while minimising adverse effects.

Moreover, SRNSs offer dual‐level control of both structure and function. These systems can be customised based on the specific pharmacological properties of the payload and optimised through multidimensional parameters, including surface‐targeting modifications, responsive threshold adjustments and multimodal coupling mechanisms, to accommodate the therapeutic demands of different OA subtypes and disease stages. For instance, in highly inflammatory OA, antioxidant drugs are preferably delivered via ROS‐responsive systems, whereas in metabolically dysregulated OA, AMPK‐activating agents are paired with visualised microenvironment‐modulating platforms to enhance the precision of personalised therapy. In this regard, SRNSs transcend their role as mere drug carriers, evolving into multifunctional therapeutic platforms that integrate lesion recognition, therapeutic amplification and signal decoding capabilities.

### 6.3 Advocating for Deep Multidisciplinary Integration Across Mechanisms, Materials and Clinical Translation

To advance SRNSs from laboratory platforms to clinically viable products, progress must extend beyond scientific feasibility to address priorities along the translational pipeline. Current experience suggests that the most critical translational bottleneck is often the establishment of GMP‐compatible manufacturability and a robust CMC (chemistry, manufacturing and controls) quality framework. Stimuli‐responsive materials frequently involve multicomponent formulations, dynamic bonds and multilayer architectures, which may lead to drift in particle size, surface chemistry and response thresholds after scale‐up. In addition, production costs and batch‐to‐batch consistency directly determine whether a product can proceed to regulatory submission and clinical manufacturing. A practical solution is to adopt a manufacturability‐driven design strategy, prioritising validated biodegradable materials and simplified structural architectures and establishing critical quality attributes and release specifications under a quality‐by‐design framework. This approach should be supported by scalable manufacturing processes and comprehensive analytical systems for stability, drug release and impurity profiling.

A second bottleneck lies in the regulatory pathway and evaluation standards for combination products, which remain incompletely harmonised. Early engagement with regulatory agencies is therefore recommended to clarify product classification, primary mechanism of action and risk categorisation. Such early alignment can help define the core safety package and CMC requirements, while also promoting the development of comparability standards, reference controls and standardised in vitro evaluation methods, thereby reducing uncertainty associated with late‐stage supplementary studies.

The third challenge concerns the generation of robust clinical evidence, particularly demonstrating advantages over conventional intra‐articular injections in heterogeneous OA populations. Feasible strategies include patient enrichment based on inflammatory phenotypes or imaging characteristics and the use of mechanism‐aligned composite endpoints, such as combining pain and functional outcomes with MRI‐based quantification of cartilage loss or synovitis, alongside fluid biomarkers. In addition, adaptive or stratified trial designs may improve statistical efficiency, while real‐world data can complement clinical trials by validating the durability of efficacy and long‐term safety.

In summary, translational progress should follow a pathway centred on manufacturability, regulatory feasibility and clinical verifiability. By prioritising manufacturing and quality systems, aligning with regulatory pathways and employing mechanism‐based clinical endpoints and patient stratification strategies, intelligent responsive systems can move beyond conceptual innovation toward practical disease‐modifying therapeutic products for OA.

### 6.4 Strategic Vision for Constructing an ‘Immunometabolic‐Delivery’ Integrated Synergistic Model

With a deeper understanding of the pathological mechanisms along the synovium‐cartilage axis in OA, growing evidence suggests that OA is not merely a degenerative condition. Instead, it represents a multicentric and dynamic disease network driven by synovial immune activation, cartilage metabolic dysregulation and joint microenvironmental imbalance. Against this backdrop, traditional single‐target interventions or linear delivery pathways can no longer meet the clinical demand for precise, effective and sustained therapies. Therefore, future efforts should focus on establishing a novel therapeutic paradigm that integrates immune recognition, metabolic modulation and delivery system synergy into a unified, closed‐loop model.

Specifically, this paradigm should encompass three core dimensions. First, at the target level, interventions should move beyond classical M1/M2 or Th17/Treg lineage modulation to encompass metabolism‐inflammation coupling targets regulated by metabolic factors such as mTOR, HIF‐1α and SIRT1. Second, at the pharmacological strategy level, synergistic multi‐molecule, multi‐mechanism regimens should be developed—for example, combining AMPK agonists, antioxidants and cartilage‐regenerative agents—to comprehensively restore microenvironmental homeostasis. Third, in delivery system design, smart nanoplatforms with sensing, responsive and adaptive capabilities must be engineered to achieve differentiated and precise drug delivery across various OA stages and tissue microdomains.

**FIGURE 15:**
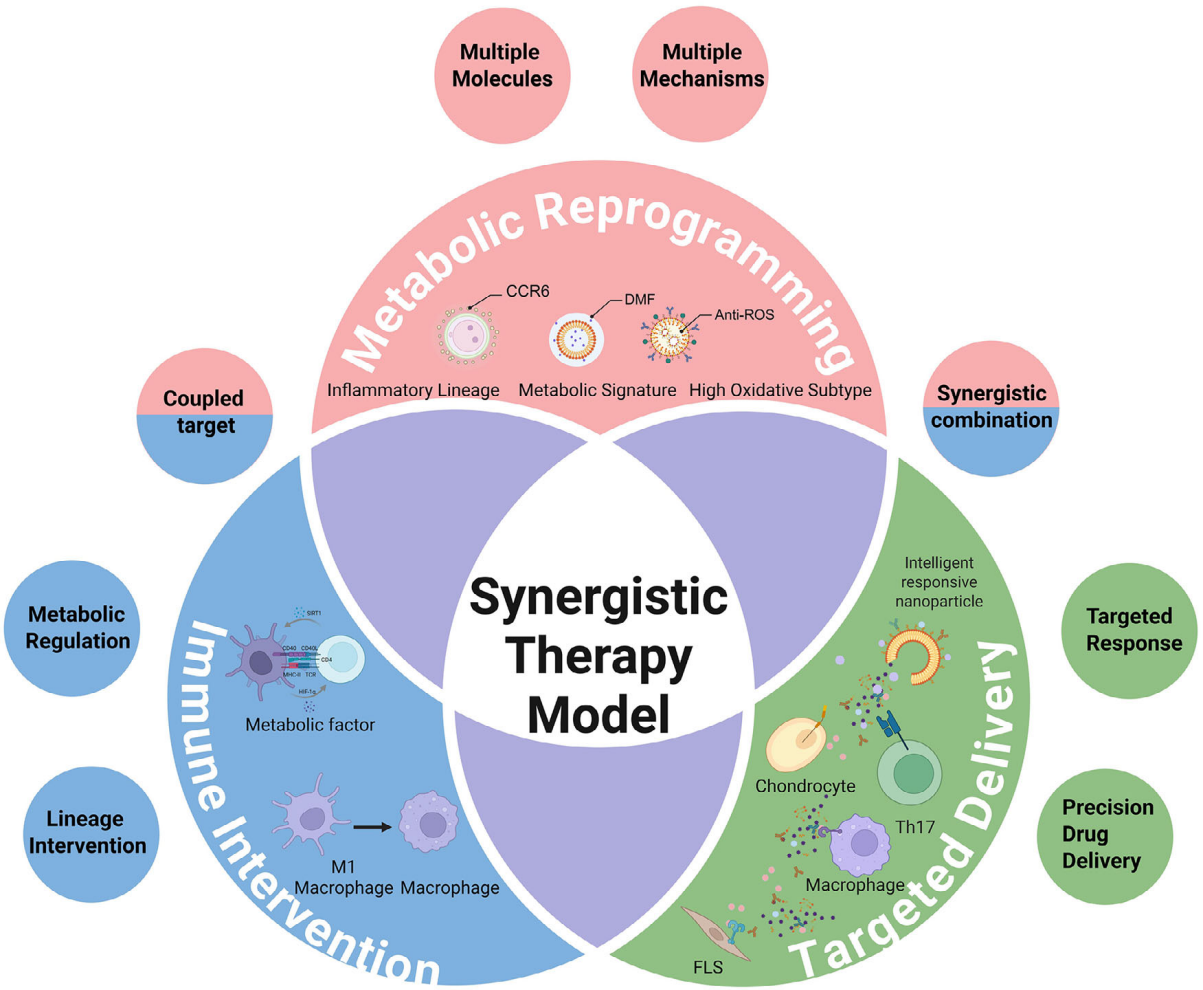
Conceptual model of an integrated ‘immunometabolic‐delivery’ tri‐modular therapeutic strategy. *Note*: CCR6, C‐C chemokine receptor type 6; DMF, dimethyl fumarate; FLS, fibroblast‐like synoviocytes; ROS, reactive oxygen species; Th17, T‐helper 17 cells.

By leveraging cross‐disciplinary technologies such as single‐cell multi‐omics, spatial transcriptomics and AI‐based computational modelling, it may become feasible to construct an integrated framework for lesion stratification, drug allocation and delivery adaptation. This collaborative model not only has the potential to transform the therapeutic landscape of OA but also offers a scalable and translatable blueprint for treating other inflammatory arthritides, such as rheumatoid arthritis and psoriatic arthritis (Figure 15).

## Author Contributions

Qirui Zhao made the major contribution to this work and is designated as the first author. Qirui Zhao, Zhewen Mi, Shuya Liu and Shijun Liang conceived the review framework and drafted the manuscript. Linjia Peng, Zixuan Gao and Jiaming Li collected and analysed the relevant literature. Xiaoqing Lu, Zhiguang Ren and Yongjie Wan contributed to data organisation and visualisation. Shengsheng Cui, Longlong Lu, Xiaoyang Gao, Tao Wang, Hui Liang, Furong Tian, de la Fuente Jesus M and Chong Xiang provided critical revisions and academic guidance. Luyi Sun, Lichen Xiang, Daxiang Cui supervised the project, provided conceptual guidance and finalised the manuscript. All authors reviewed and approved the final version of the manuscript.

## Conflicts of Interest

The authors declare no conflicts of interest.

## Data Availability

All data generated or analysed during this study are included in this article and/or its supplementary material files. Further enquiries can be directed to the corresponding author.
